# The Functional and Regulatory Mechanisms of the *Thellungiella salsuginea* Ascorbate Peroxidase 6 (TsAPX6) in Response to Salinity and Water Deficit Stresses

**DOI:** 10.1371/journal.pone.0154042

**Published:** 2016-04-20

**Authors:** Zeqin Li, Jilong Zhang, Jingxiao Li, Hongjie Li, Genfa Zhang

**Affiliations:** 1 Beijing Key Laboratory of Gene Resource and Molecular Development, College of Life Sciences, Beijing Normal University, Beijing, China; 2 The National Key Facility for Crop Gene Resources and Genetic Improvement, Institute of Crop Science, Chinese Academy of Agricultural Sciences, Beijing, China; Texas Tech University, UNITED STATES

## Abstract

Soil salinization is a resource and ecological problem in the world. *Thellungiella salsuginea* is becoming a new model plant because it resembles its relative species, *Arabidopsis thaliana*, in small genome and short life cycle. It is highly tolerant to salinity and drought stresses. Ascorbate peroxidase (APX) is an enzyme that clears H_2_O_2_ in plants. The function and molecular and regulation mechanisms of APX in *T*. *salsuginea* have rarely been reported. In this study, an APX gene, *TsApx6*, was cloned from *T*. *salsuginea* and its responses to abiotic stresses in transgenic *Arabidopsis* were studied. Under high salinity treatment, the expression of *TsApx6* was significantly induced. Under drought treatment, overexpression of *TsApx6* increased the survival rate and reduced leaf water loss rate in *Arabidopsis*. Compared to the wild type plants, high salinity treatment reduced the concentrations of MDA, H_2_O_2_ and proline but elevated the activities of APX, GPX, CAT and SOD in the *TsApx6*-overexpressing plants. Meanwhile, germination rate, cotyledon greening, and root length were improved in the transgenic plants compared to the wild type plants under salt and water deficit conditions. Based on these findings, *TsApx6* has an important function in the resistance of plants to certain abiotic stresses. The *TsApx6* promoter sequence was obtained using Genome Walking technology. Bioinformatics analysis indicated that it contains some *cis*-acting elements related to stress response. The treatments of salt, dehydration, and ABA induced the expression of *Gus* gene under the regulation of the *TsApx6* promoter. Mutation analysis showed that the MBS motif present in the *TsApx6* promoter might be a key negative regulatory element which has an important effect on the growth and developmental process of plants.

## Introduction

The life cycle of a plant is influenced by various environmental factors. Drought and salinity are important in reducing plant productivity and limiting plant distribution. Although drought stress is more pervasive and economically damaging than salt stress, many studies on water stress focused on the effects of salt treatment. This lies in the fact that salt stress can be realized easily in a controlled condition and the responses of a plant to drought and salt stresses are associated with each other [1]. When grown under saline and drought conditions, plants produced excess reactive oxygen species (ROS), which include hydrogen peroxide (H_2_O_2_), singlet oxygen (^1^O_2_), hydroxyl radicals (•OH), and superoxide radicals (•O_2_^-^) in cells [2–4]. Reactive oxygen species have a key function to serve as signaling molecules in regulating growth, development, acclimation to abiotic stresses, and initiating pathogen defense pathways. However, over-accumulation of ROS resulted in oxidative damage to different cellular components [3,5–7]. Plants have a large network for ROS production and scavenging in various cellular compartments that are essential for regulating ROS signals in the growth, development, and defense against environmental stresses. In *Arabidopsis thaliana* L., such a network consists of at least 152 genes encoding ROS producing proteins, including nicotinamide adenine dinucleotide phosphate (NADPH) oxidases and ROS removing enzymes. This class of enzymes includes ascorbate peroxidases (APX, EC 1.11.1.1), glutathione peroxidase (GPX, EC 1.11.1.9), catalase (CAT, EC 1.11.1.6) and superoxide dismutases (SOD, EC 1.15.1.1) [8–11]. Ascorbate peroxidases with the strongest affinity for H_2_O_2_ are central components of the H_2_O_2_ scavenging networks and very important in the regulation of cellular ROS levels [12].

An *Arabidopsis* APX family, which consists of eight enzymes, uses ascorbic acid (AsA) as the substrate to reduce H_2_O_2_ into water [8,13]. Among the three cytosolic members (APX1, APX2 and APX6) in *Arabidopsis*, the functions of APX1 and APX2 are well studied [13–16]. As the most abundant APX member, APX1 is involved in many biological processes. This enzyme is expressed in different organs of plants, for example, root, stem, and leaf. The expression of APX1 is significantly promoted by different biotic and abiotic stresses [14,17,18]. The knockout plant that is deficient in APX1 had high accumulation of H_2_O_2_ under various growth conditions. Such a plant also had oxidized proteins under light stress, high sensitivity to oxidative stress, and growth suppression [14,17]. Cytosolic APX1 proved to be effective in protecting the chloroplasts during an excessive radiation and thylakoids and stromal/mitochondrial APXs [14]. These findings indicate that the APX1 has a function in preventing cellular components from oxidative damage and regulating cellular and intracellular ROS signals.

Under normal conditions, the expression level of *Apx2* is extremely low so that it is undetectable in most plant tissues. However, upregulation of *Apx2* can be promoted by wounding, heat, osmotic and oxidative stresses in roots, heat, saline and drought stresses in shoots, and heat treatment in leaves and pollen [18,19]. The expression of *Apx2* gene is upregulated as high as approximately 10 folds by the drought stress and the high irradiance, about 20 folds by the treatment of H_2_O_2_, and over 20 folds by the heat shock [13,20,21]. Heat stress also significantly upregulates the expression of tomato (*Solanum lycopersicum* L.) *Apx2* gene in pollen [19]. The lack of APX2 ensures the *Arabidopsis* plants more sensitive to the heat stress at the seedling stage, but makes them more tolerant to the heat stress at the reproductive stage compared to the wild-type (WT) plants [15]. These findings suggest that APX2 might have a function in the regulation of tolerance to heat at different developmental stages in plants.

*Arabidopsis thaliana* is a model plant in the studies of the molecular basis of tolerance to various stresses [1,22]. Because many knock-out mutants are available and genetic transformation is amenable, it is readily to dissect the functions of different stress associated genes using *A*. *thaliana*. However, it is difficult to fully understand the salt-tolerant mechanisms only by studying *A*. *thaliana*, a true glycophyte. *Thellungiella salsuginea* O.E. Schulz is a salt-tolerant plant species that can serve as an informative model to unravel the nature of tolerance against saline stress. As a close relative of *A*. *thaliana*, *T*. *salsuginea* shares 92% nucleotide similarity at the cDNA level with *Arabidopsis*. This makes it possible to use the resources of *Arabidopsis*, such as the information on genes and proteins, in the studies of *T*. *salsuginea*. *Thellungiella salsuginea* plants are extremely tolerant to salt stress, which can survive in seawater and reproduce under the salt stress as high as 500 mM NaCl treatment [23]. *Thellungiella salsuginea* also tolerates to ozone, drought, heat, freezing, and chilling [24]. In addition to these abiotic stresses, *T*. *salsuginea* has numerous desirable traits, for example, small genome size, short plant height and life cycle, self-fertility, and plenty of seeds. All these attributes make it suitable as a promising halophytic model plant in the genetic and genomic studies [23]. The isolation of candidate genes for salt tolerance has provided new information on the mechanisms of salt tolerance in *T*. *salsuginea* [24]. Nevertheless, the mechanisms underlying the tolerance to salt and other abiotic stresses are not fully understood.

APX6, the third cAPX of the *Arabidopsis*, protects mature desiccating and germinating seeds from excessive oxidative stress and maintains seed vigor in front of stress conditions [16]. Nevertheless, the expression and other functions of APX6 in the acclimation of plants to biotic and abiotic stress conditions are not known. In the present study, *TsApx6* gene was cloned from *T*. *salsuginea* and the expression patterns under a high level of salt treatment was studied. The transgenic *A*. *thaliana* lines that constitutively overexpressed *TsApx6* were produced. Using these transgenic lines, the concentrations of H_2_O_2_, proline and malondialdehyde (MDA), as well as the antioxidant enzyme activities under the high salt stress treatment were determined. The *A*. *thaliana* transformed plants carrying *TsApx6* in the *atapx6* loss-of-function mutant were also studied. In addition, the promoter regions of *TsApx6* were cloned and analyzed. In the transgenic *Arabidopsis* plants, the *Gus* reporter gene, which was driven by the *TsApx6* promoter, was induced by salt stress, in particular in the seedlings and flowers. By analyzing the sequences and MBS (a MYB binding site) element mutants, we identified a *cis*-acting element from the *TsApx6* promoter, which may be a key negative regulatory element for accurately regulating the process of growth and development and responding to the environmental stresses.

## Materials and Methods

### Plant materials, growth conditions and abiotic stress treatments

The ecotype Shandong of *T*. *salsuginea* and the ecotype Columbia (Col-0) of *A*. *thaliana* were used throughout the study. A line of *atapx6* T-DNA insertion mutant (WiscDsLox321c09) was provided by the Arabidopsis Biological Resource Center (ABRC, http://abrc.osu.edu/). All the transgenic plants were produced in the background of Col-0, except for the *atapx6/35S*:*TsApx6-GFP* line in which the *TsApx6* (GenBank Accession No. AK353188) was transformed into the *atapx6* homozygous mutant. Seeds were sterilized using 0.1% (w/v) HgCl_2_ solution and sown on the Murashige and Skoog (MS) medium (pH 5.8) supplemented with 3% (w/v) sucrose and solidified with 0.7% (w/v) agar. Germination, cotyledon greening and root growth assay were performed under various concentrations of NaCl (0, 50, 100 and 150 mM) or mannitol (0, 100, 200 and 300 mM). The surface-sterilized seeds were stratified at 4°C in the dark for 3 d prior to transferring to a growth chamber, which was set at 22°C with a 16-h light (110 μmol m^-2^ s^-1^) and 8-h dark photoperiod and a relative humidity of 70%. Alternatively, they were directly sown in pots filled with a soil-Vermiculite mixture (3:1) after stratification under the same conditions. The rates of radicle appearance and cotyledon greening were determined at 3 d and 7 d after they were cultured, respectively. To measure root growth, seedlings (3-d-old) grown on MS medium were transferred onto new MS plates supplemented with appropriate concentrations of NaCl or mannitol. After 10 d of growth on new medium, root length of seedlings was measured.

To investigate the expression profiles of *TsApx6* and *AtApx6* (GenBank Accession No. NM_119384) under short-term of NaCl treatment, wild-type *Thellungiella* and *Arabidopsis* seedlings at the 4–6 leaf-stage were transferred to the plastic pots containing a Vermiculite and Perlite mixture (3:1, v/v) and watered with the Hoagland nutrient solution supplemented with 300 mM NaCl for 0, 2, 4, 6, 8, 10, 12, 24, 36 or 48 h [25]. To determine the salt-tolerance of the transgenic genotypes, plants were grown in the soil and Vermiculite mixture (3:1, v/v) for four weeks before they were treated with 300 mM NaCl solution for 3 d, and the leaves were harvested from 5 plants of each line. The concentrations of H_2_O_2_, proline and MDA and the activities of APX, GPX, CAT and SOD enzymes in the transgenic and non-transgenic lines were measured using the methods that were described previously [26]. Each experiment was carried out at least in triplicate. To determine the expression of the stress/ABA-responsive genes, the WT and the *35S*:*TsApx6-GFP* transgenic *Arabidopsis* plants were immersed in 300 mM NaCl for 6 h and 72 h, respectively.

For measurement of drought tolerance, watering was stopped from the 4-week-old plants for 20 days before resuming watering. Photographs were taken and the survival rates of plants were determined 3 d after recovery. Water status for the leaf tissue was determined by the water loss rate, and the detail method was described previously [26].

For GUS activities assay under abiotic stresses, NaCl, mannitol and abscisic acid (ABA) treatments were applied by immersing the seedlings one-week-old, leaves from plants three-week-old and flowers from plants five-week-old in MS nutrition solutions supplemented with 200 mM NaCl, 300 mM mannitol or 0.1 mM ABA for 10 h, respectively.

### Gene expression analysis by real-time quantitative PCR

Total RNA was extracted from the plant tissues grown in the control or various stress conditions using an RNeasy kit (Qiagen, Amsterdam, Netherlands). One μg of RNA from each sample was used in the reverse transcription with a cDNA synthesis kit (Code No.: AH311-02, TransGen, Beijing, China) according to the instructions provided by the manufacturer. Real-time quantitative PCR (qPCR) was conducted on an ABI 7500 system (Applied Biosystems, New York, USA) under the default thermal cycling conditions using a TransStart^TM^ Green qPCR SuperMix kit (TransGen) and specific primers for PCR amplification. Relative transcript abundance was determined and normalized with the reference gene *Actin2* (GenBank Accession No. U41998) to minimize variation in the levels of the cDNA templates. For expression profile examination of *TsApx6*, *AtApx6* and stress-related genes under salt stress conditions, the control sample acted as the calibrator with a nominal value of 1. When investigated the *Gus* expression, *Actin2* itself was calibrated with a nominal value of 100. The data represent mean values derived from at least three independent repetitive amplifications and each experiment was conducted in at least three biological replicates. All calculations and analyses were performed by the 2^-△△Ct^ method. The primers used in qPCR of *TsApx6*, *AtApx6*, *Actin2* and the stress/ABA-responsive genes are shown in Table A in S1 Text.

### Construction of plasmids and generation of transgenic plants

The *TsApx6* and *AtApx6* cDNAs were amplified using the primers *Apx6 Pst* I-LP and *Apx6 Avr* II-RP (Table A in S1 Text) for their identical primer sequences. The PCR products were digested by *Pst* I and *Avr* II and cloned into the modified binary vector pCAMBIA1302 that was digested with the same enzymes to generate 35S:*TsApx6*/*AtApx6*-*GFP* fusion construct. For the *TsApx6* and *AtApx6* promoter (containing the *TsApx6* promoter whose MBS motif was mutated) and *Gus* fusion construct, the 5' flanking sequences were amplified from the genomic DNA with the appropriate forward and reverse primers that contained *Sal* I in the 5' end and *Spe* I in the 3' end, as shown in Table A in S1 Text (Ts6P-LP, Ts6P-RP, At6P-LP and At6P-RP). Then, the fragments generated were cloned into the appropriate sites located before the *Gus* reporter gene in the binary vector pCAMBIA1305.1. All the plasmids constructed were introduced into *Agrobacterium tumefaciens* Smith & Townsend strain GV3101 to deliver them into the wild-type *Arabidopsis* by means of the floral-dip method. Complementary transgenic plants of *TsApx6* were produced by introducing a 35S:*TsApx6*-*GFP* construct into the *atapx6* knockout mutant background. The transgenic lines were selected with 40 μg mL^-1^ hygromycin and confirmed by PCR amplification using the specific primers listed in Table A in S1 Text. Generation T3 plants carrying a homozygous single insertion were selected for further detailed analysis. For analyzing the phenotypic and physiological properties, *35S*:*TsApx6-GFP* (lines 2 and 4), *35S*:*AtApx6-GFP* (lines 8 and 12), and *atapx6/35S*:*TsApx6-GFP* (lines 1 and 6) were applied.

### Isolation and bioinformatics analysis of promoter sequences

A 1,654 bp fragment located in the upstream of the ATG start codon of *TsApx6* was amplified using the genomic DNA isolated from *T*. *salsuginea* by chromosome walking using a Genome Walking kit (Code No.: 6108, TaKaRa, Dalian, China) and gene specific SP primers Ts6P-1SP1, Ts6P-1SP2, Ts6P-1SP3, Ts6P-2SP1, Ts6P-2SP2, and Ts6P-2SP3 (Table A in S1 Text). The orthologous gene of *TsApx6* in *Arabidopsis* is *AtApx6* (AT4G32320). The same size upstream flanking region of *AtApx6* was amplified with primers At6P-LP and At6P-RP (Table A in S1 Text) using the *Arabidopsis* genomic DNA as template, whose sequence was provided by The Arabidopsis Information Resource (TAIR) database (http://www.arabidopsis.org). Sequence alignment of the two promoters was performed against the database of the National Center for Biotechnology Information (NCBI, http://www.ncbi.nlm.nih.gov/), and the regulatory elements distributed on them were analyzed using the program PLANTCARE, which is a database of plant *cis*-acting regulatory elements, enhancers and repressors (http://bioinformatics.psb.ugent.be/webtools/plantcare/html/). MBS is a *cis*-acting regulatory element that was predicted to be a MYB binding site involved in drought inducibility. In order to confirm experimentally the function of MBS, we obtained a new promoter sequence (designated Ts6P-M_0_) on which the MBS motif was changed. In the new sequence, the thymine (T) of the MBS motif was deleted and one cytosine (C) was replaced by adenine (A). The specific process is shown in S1 Fig.

### GUS histochemical staining and fluorometric assay

The activities of GUS in the *Arabidopsis* transgenic lines were determined using one-week-old seedlings, leaves from three-week-old plants, flowers, stems and siliques from five-week-old plants. Plant tissues were treated in 90% acetone for 30 min, washed with 50 mM sodium phosphate buffer (pH 7.2) for three times, and then incubated at 37°C overnight in a GUS staining solution (50 mM, pH 7.2), which consisted of 0.1% Triton X-100, 1 mM X-Gluc, 2 mM potassium ferrocyanide, 2 mM potassium ferricyanide and 10 mM ethylene diamine tetraacetic acid (EDTA, pH 8.0). The tissues stained were immersed in 70% ethanol for several hours to remove the chlorophyll before observing under a stereo fluorescence microscope (Discovery V12, Zeiss, Jena, Germany).

For quantitative fluorometric GUS assay, approximate 0.1 g of plant tissues were chilled in liquid nitrogen and ground in GUS extraction buffer (10 mM EDTA, pH 8.0, 50 mM sodium phosphate buffer, pH 7.0, 0.1% β-mercaptoethanol, 0.1% sodium dodecyl sulfonate (SDS), 0.1% Triton X-100 and 20% methanol). The extracts were centrifuged at 4000 ×g for 10 min to produce the supernatants for GUS assays. The protein concentration was determined as described previously [27]. The activity of GUS was determined using 4-methylumbelliferyl-β-D-glucuronide as the substrate following the method described by Jefferson [28]. The reaction was stopped by adding 200 mM Na_2_CO_3_. Fluorescence was quantified using a FluoroMax-4 fluorescence photometer (HORIBA Jobin Yvon, Paris, France) with the excitation and the emission filters at 365 nm and 455 nm, respectively.

### Statistical analysis

All above experiments were repeated at least three times, and all the data are presented as means ± standard deviations (SD). One-way analysis of variance (ANOVA) was performed for all the data and the significance of difference in values (*P* < 0.05 or *P* < 0.01) was determined by the Fisher’s least significant difference (LSD) test using SPSS 20.0 for Windows. All figures were made with the Sigma Plot 12.5 software.

## Results

### Isolation and sequence analysis of *TsApx6* from *T*. *salsuginea*

The *TsApx6* cDNA (GenBank Accession No. AK353188) was 1,109 bp in length. It consists of a 987 bp open reading frame (ORF), encodes an APX enzyme of 328 amino acids, and has the predicted molecular mass of 36.4 kDa. Result of protein database search (http://ipsort.hgc.jp/index.html) showed that there was no nuclear localization signal, chloroplast transit, or mitochondrial targeting sequence in the N-terminus of TsAPX6 protein. This indicates that this protein might locate in the cytoplasm. Evolutionary relationship of TsAPX6 from *T*. *salsuginea* with other APXs from different plant species was detected by the phylogenetic analysis. Specially, this protein showed the highest similarity with *Arabidopsis* APXs and exhibited 86% identity with AtAPX6 (S2 Fig).

### Expression profile analysis of *TsApx6* under NaCl treatment

**Fig 1:**
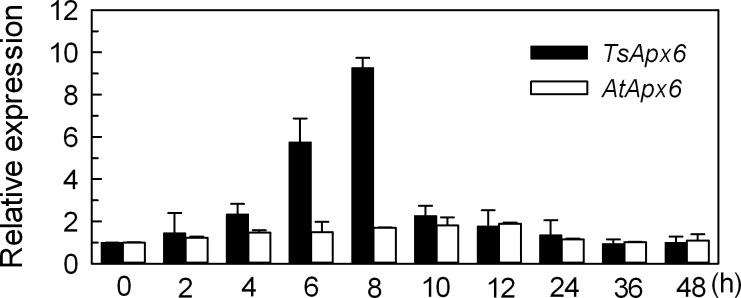
Expression of *TsApx6* and *AtApx6* under the salt stress conditions as revealed by qPCR analysis. *Thellungiella* plants (six-week-old) and *Arabidopsis* plants (four-week-old) were treated with 300 mM NaCl for 0, 2, 4, 6, 8, 10, 12, 24, 36, or 48 h, respectively. Data are shown as the means ± standard deviation (SD) from three replicates.

To examine whether *TsApx6* expression is stress inducible, qPCR analysis was conducted using total mRNA purified from the leaves of plants (6-week-old) grown under 300 mM NaCl treatment or normal condition. The *TsApx6* mRNA levels increased rapidly in the case of 300 mM NaCl treatment by about 10-fold at 8 h (*P* < 0.01), whereas *AtApx6* mRNA showed a 1.9-fold induction at 12 h by the treatment of NaCl (*P* < 0.05) (Fig 1). This result suggests that *TsApx6* has an important function in the response to salt stress in *T*. *salsuginea*.

### Expression of *TsApx6* is closely associated with development of plants at the germination and post-germination stages in the presence of NaCl or mannitol

**Fig 2:**
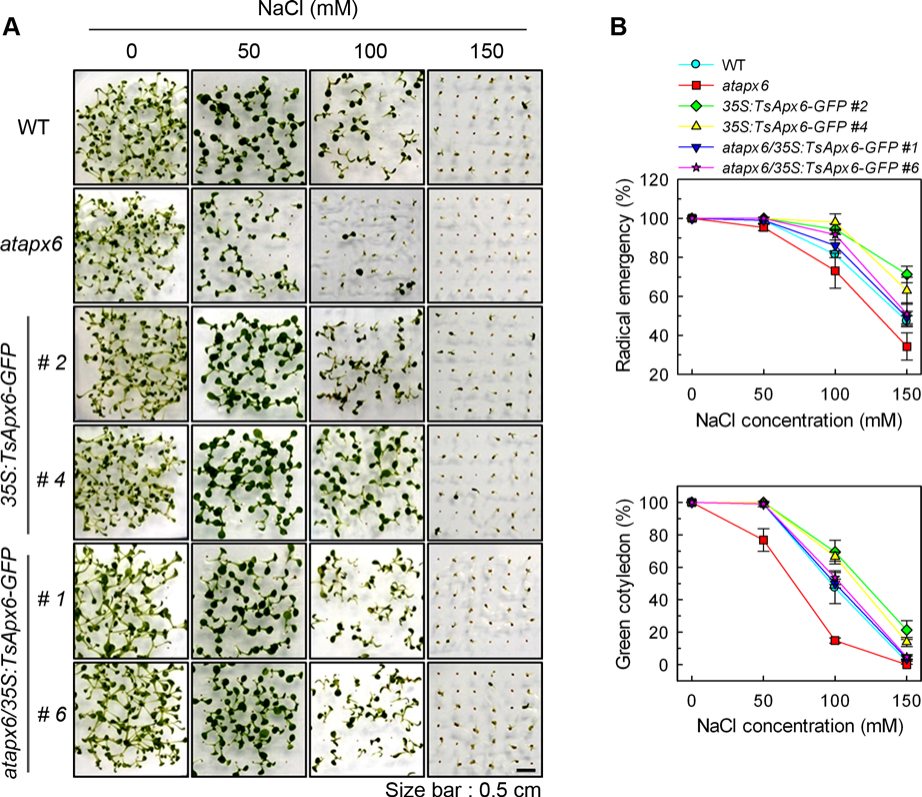
Phenotypes of each line in the presence of NaCl treatment during seed germination and early growth. (A) Sensitivity to NaCl in the WT, *atapx6*, and *TsApx6* transgenic plants during seed germination. Surface-sterilized seeds were sown on MS media contained 0, 50, 100 or 150 mM NaCl, and incubated at 22°C for 7 d under a 16-h light and 8-h dark photoperiod. Size bar = 0.5 cm. (B) Quantification of radicle appearance at 3 d after sowing and cotyledon greening at 7 d after sowing in response to NaCl. Data represent means ± SD from four biological replicates (n = 144).

**Fig 3:**
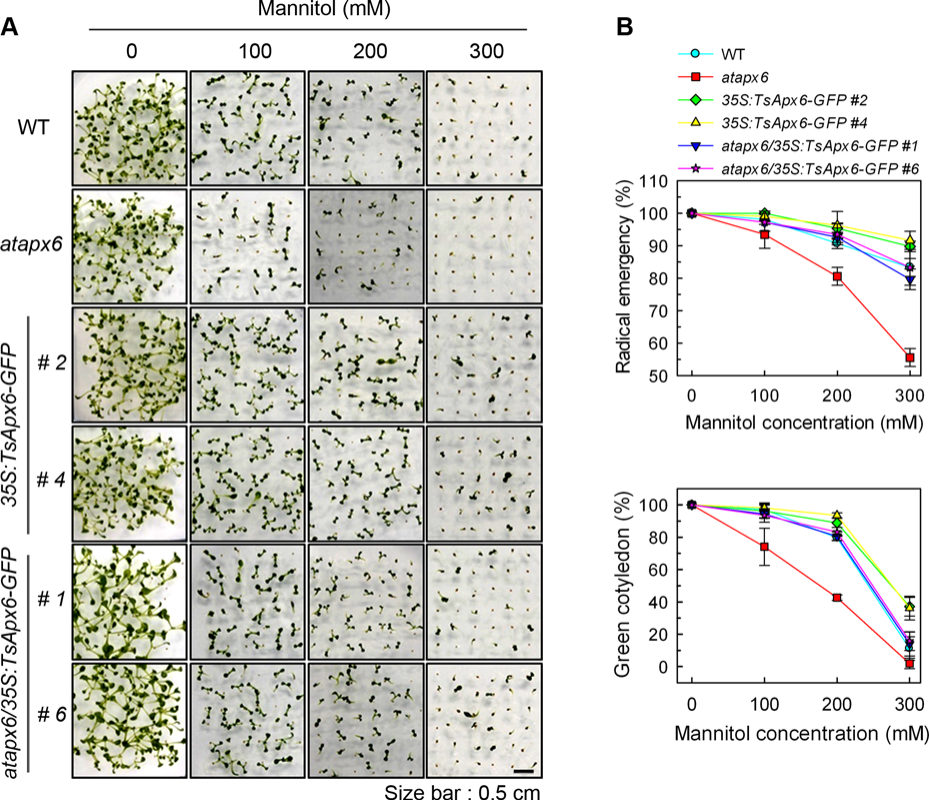
Phenotypes of each line in the presence of mannitol treatment during seed germination and early growth. (A) Mannitol sensitivity of all lines at the germination stage. Surface-sterilized seeds were sown on MS medium supplemented with 0, 100, 200, or 300 mM mannitol, and incubated at 22°C for 7 d under a 16-h light and 8-h dark photoperiod. (B) Quantification of radicle emergence at 3 d after sowing and cotyledon greening at 7 d after sowing in response to mannitol. Data represent means ± SD from four biological replicates (n = 144).

The *atapx6* mutant was chosen based on its mannitol and NaCl-sensitive phenotype. Germination rates for seeds of the *atapx6*, WT, *atapx6/35S*:*TsApx6-GFP*, and *35S*:*TsApx6-GFP* were compared under the treatments of 50, 100, and 150 mM of NaCl or 100, 200, and 300 mM of mannitol. On the growth media containing NaCl or mannitol, seed germination rates decreased with the increase of the concentrations of NaCl or mannitol. When treated with 100 mM NaCl, the *atapx6* T-DNA insertion mutant seeds displayed a more sensitive phenotype to NaCl compared to the WT seeds (*P* < 0.01), 19% of the WT seeds did not germinate at 3 d after culture, and greater than 27% of the seeds from the mutant didn’t germinate normally. In contrast, both *atapx6/35S*:*TsApx6-GFP* and *35S*:*TsApx6-GFP* transgenic seeds were highly resistant to NaCl. Over 50% of those transgenic seeds germinated under a high NaCl concentration (150 mM) (Fig 2B). Mannitol had different effects on the radicle emergence of seeds on the NaCl-containing medium. The germination rates for the wild-type and the transgenic seeds were over 80% under 300 mM mannitol treatment, with the exception of *atapx6* (55%) (*P* < 0.01) (Fig 3).

Moreover, at 7 d after germination, the growth of 85% of *atapx6* seedlings was fully inhibited by 100 mM NaCl treatment, and the green cotyledons failed to develop. In contrast, approximately 52% of *atapx6/35S*:*TsApx6-GFP* and 67% of *35S*:*TsApx6-GFP* overexpressing genotypes developed green cotyledons under the same concentration of NaCl (*P* < 0.01) (Fig 2B). Mannitol had similar effects on NaCl treatment in terms of the cotyledon greening. Growth of almost all the *atapx6* seedlings was inhibited by 300 mM mannitol treatment and only 11% of the WT plants developed true green cotyledons (*P* < 0.01) (Fig 3). However, over 14% of *atapx6/35S*:*TsApx6-GFP* and 36% of *35S*:*TsApx6-GFP* overexpressing lines produced green cotyledons under the same concentration of mannitol (*P* < 0.01). This result indicates that *TsApx6*-overexpressing lines are more tolerant to salt and osmotic stresses.

**Fig 4:**
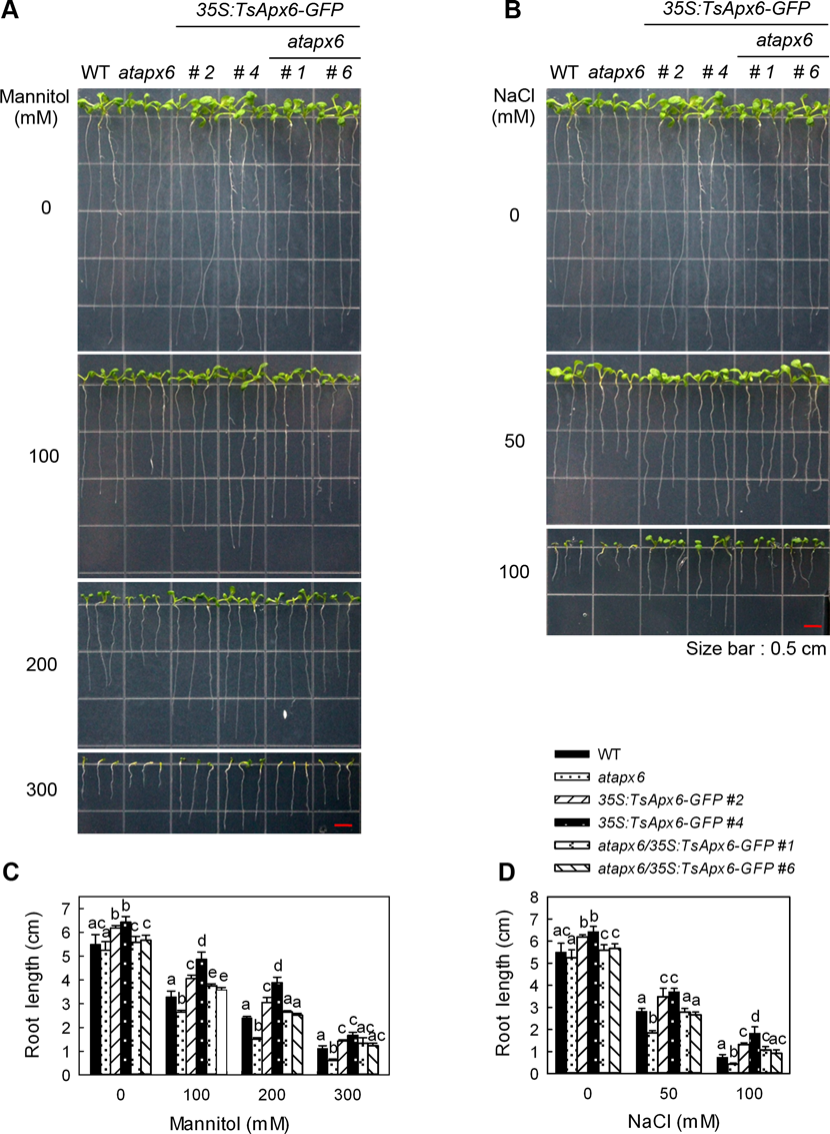
Root growth of all lines in response to different concentrations of NaCl or mannitol. Surface-sterilized seeds were germinated on full-strength MS medium for 3 d and the seedlings were then moved to fresh MS medium that contained 0, 50 or 100 mM NaCl or 100, 200 or 300 mM mannitol and grown vertically for 10 d. Bars = 0.5 cm. The data represent the means ± SD (n = 45). Means followed by different letters are significantly different at *P* < 0.05.

Salt and osmotic stresses produced obvious effects on the root growth on the tenth day after germination in the *atapx6*, WT, *atapx6/35S*:*TsApx6-GFP*, and *35S*:*TsApx6-GFP* plants. The difference in phenotype and growth values was significant among *35S*:*TsApx6-GFP* plants and other lines even though they were normally grown (*P* < 0.05) (Fig 4). The root elongation of all lines was significantly limited even by a low concentration of NaCl (50 mM) or mannitol (100 mM), indicating an increase in the sensitivity to NaCl and mannitol treatment (Fig 4). With the increase of salt or mannitol concentrations, root elongation decreased in all lines especially in the *atapx6* loss-of-function mutants (Fig 4). On the MS medium supplemented with 100 mM NaCl or 300 mM mannitol, the root growth of *atapx6* plants was greatly inhibited, which was different from the *35S*:*TsApx6-GFP* roots that elongated under the same conditions (*P* < 0.01). The root growth of WT and *atapx6/35S*:*TsApx6-GFP* lines were in the middle between the lines of *atapx6* and *35S*:*TsApx6-GFP* (Fig 4). Thus, the *atapx6* mutant was sensitive to salt and osmotic stresses in terms of root growth. In contrast, the roots of *35S*:*TsApx6-GFP* (lines 2 and 4) overexpressing lines displayed lower sensitivity to NaCl and mannitol (*P* < 0.01) (Fig 4). These results indicate that the expression of *TsApx6* was closely associated with the root development during germination (Figs 2 and 3) and post-germination (Fig 4) stages in *Arabidopsis*.

### *Arabidopsis* plants overexpressing *TsApx6* are more resistant to high salinity stress than the WT genotypes

**Fig 5:**
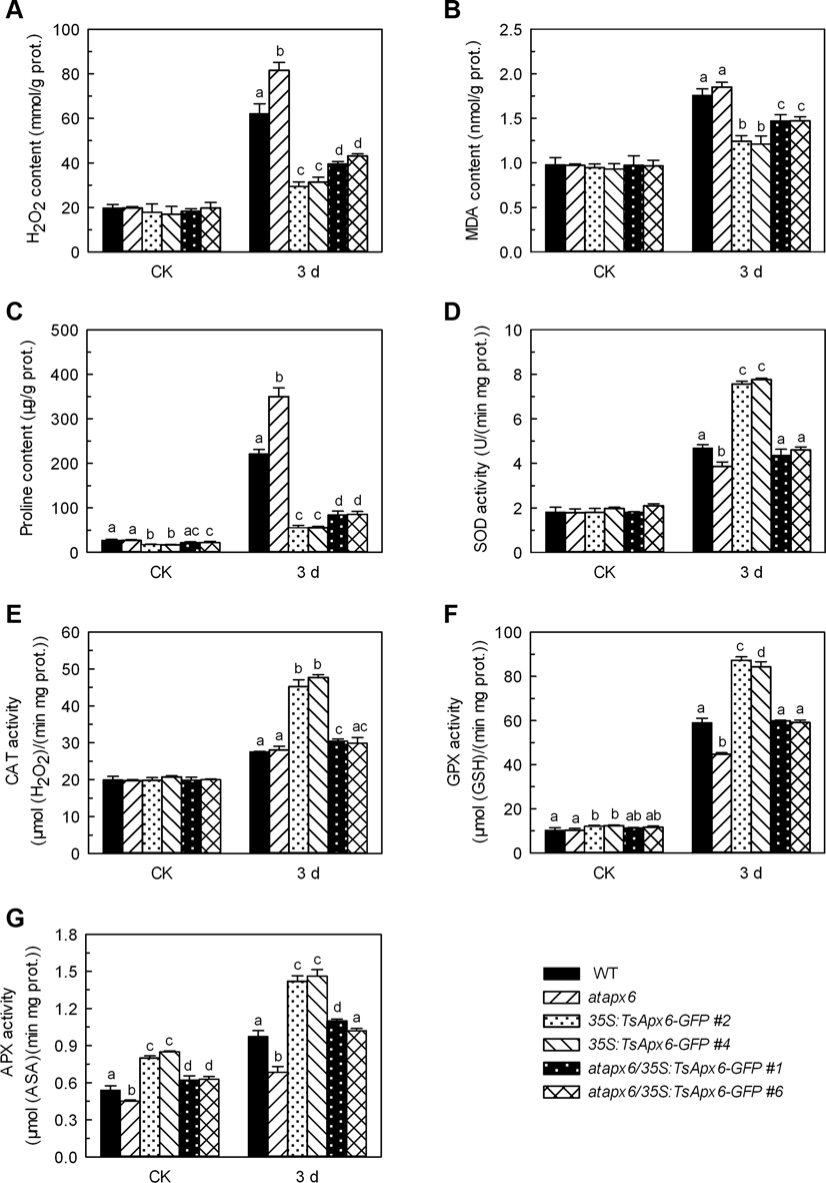
Effects of high salinity stress on the MDA, H_2_O_2_ and proline concentrations and APX, GPX, CAT and SOD activities in plants. Four-week-old wild-type, *atapx6* mutant, *atapx6/35S*:*TsApx6-GFP* and *35S*:*TsApx6-GFP* plants were treated with 300 mM NaCl and the leaves were harvested after 3 d of the treatment. A significant difference (*P* < 0.05) among the genotypes was shown by different letters (a–d). There was no statistic difference among the groups that are not marked with letters. All data were shown as the means ± SD from three biological replicates (n = 30).

To investigate whether constitutive expression of the *TsApx6* gene protects *Arabidopsis* from high salinity stress, the concentrations of H_2_O_2_, MDA and proline and the activities of APX, GPX, CAT and SOD in the *atapx6*, WT, *atapx6/35S*:*TsApx6-GFP* and *35S*:*TsApx6-GFP* lines were measured before and after the treatment by 300 mM NaCl for 3 days. Before salt treatment, the concentration of H_2_O_2_ did not differ significantly among the WT, *atapx6* mutant, and the transgenic lines (Fig 5A). After the NaCl treatment, the H_2_O_2_ concentration significantly increased in all lines particularly in the non-transgenic plants (*P* < 0.05). The amount of H_2_O_2_ in the *atapx6* mutant was 1.31, 1.99, and 2.72 folds greater than that of the WT, *atapx6/35S*:*TsApx6-GFP*, and *35S*:*TsApx6-GFP* genotypes, respectively (Fig 5A). This suggests that *TsApx6* is involved in salt stress induced H_2_O_2_ elimination in the transgenic *Arabidopsis*. Malondialdehyde was highly accumulated in all plants after salt treatment for 3 days, indicating the occurrence of the lipid peroxidation caused by salt stress in all lines. However, the MDA concentration was significantly greater in the WT and *atapx6* mutant than in the *35S*:*TsApx6-GFP* overexpressing lines (*P* < 0.01), and the MDA concentration of *atapx6*/*35S*:*TsApx6-GFP* was smaller than that of the WT but greater than that of the *35S*:*TsApx6-GFP* transgenic plants (*P* < 0.05) (Fig 5B). These results indicated that *TsApx6* overexpressing plants had a better protection against oxidative damage and might be salt tolerant. Under the normal growth condition, a significant difference in proline levels was observed between the *TsApx6*-overexpressing and other plants (*P* < 0.05) (Fig 5C). Under the high salinity condition, the proline concentration was significantly greater in the *atapx6* mutant than in the WT (*P* < 0.01); however, it was significantly smaller in the *atapx6/35S*:*TsApx6-GFP* and the *35S*:*TsApx6-GFP* transgenic genotypes than in the WT (*P* < 0.01) (Fig 5C). In addition, we found that prior to NaCl treatment, no obvious difference for SOD and CAT activities was observed among all lines (Fig 5D and 5E). The GPX activities were greater in the *35S*:*TsApx6-GFP* transgenic lines than in the WT and the *atapx6* loss-of-function mutant (*P* < 0.05), and the APX activities in the *35S*:*TsApx6-GFP* transgenic lines were significantly greater than that of the other lines (*P* < 0.05) (Fig 5F and 5G). Salinity stress induced the greatest activities of APX, GPX, CAT and SOD in the leaves of all NaCl treatment groups. The activities of the antioxidant enzymes displayed similar patterns in responses to salt stress (Fig 5D–5G). The activities of all the antioxidant enzymes in transgenic lines, especially *35S*:*TsApx6-GFP*, were prominently greater than those in the *atapx6* mutant and the WT (*P* < 0.01). These findings strongly indicated that the expression of *TsApx6* in *Arabidopsis* resulted in the coordinated increase of antioxidant enzyme activities under the conditions of salt stress, which provided a common protection for plants against oxidative damage.

### Overexpression of *TsApx6* improves tolerance of *Arabidopsis* to drought stress

**Fig 6:**
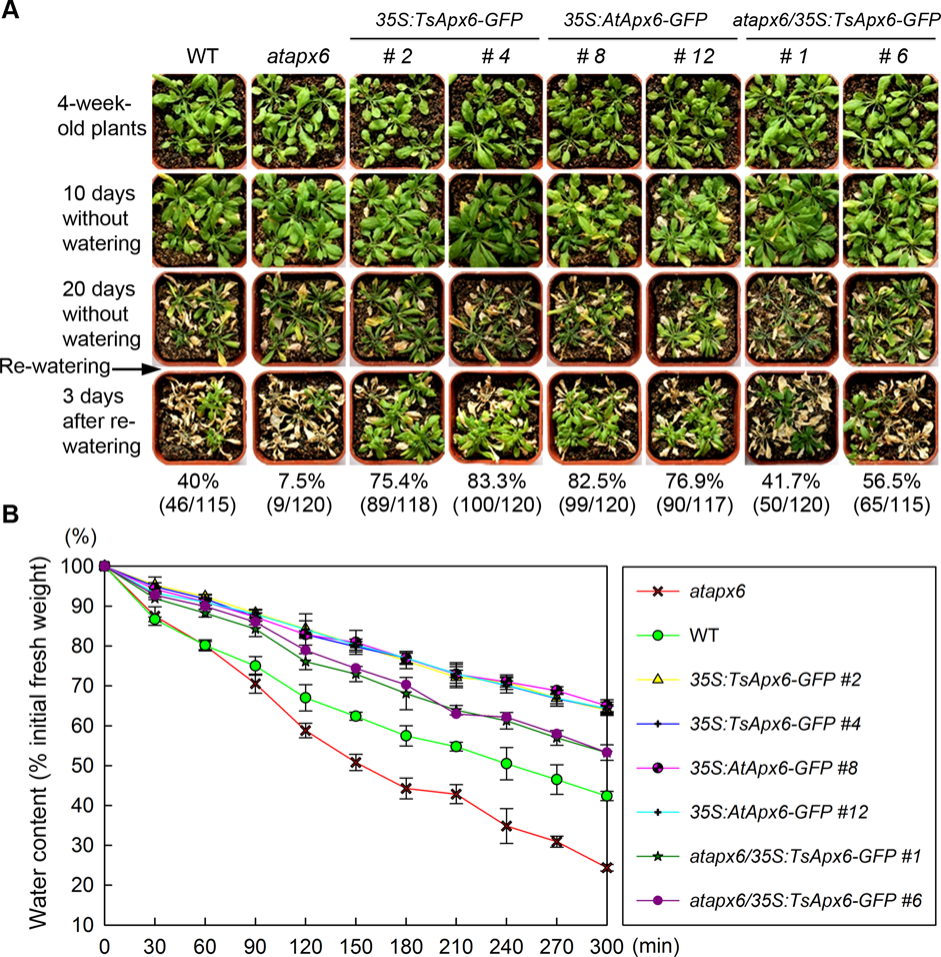
Estimation of drought stress resistance of the *TsApx6*-overexpressing plants. (A) Drought tolerance of the WT, *atapx6*, *atapx6/35S*:*TsApx6-GFP*, *35S*:*TsApx6-GFP*, and *35S*:*AtApx6-GFP* plants. Plants (four-week-old) were subjected to drought stress by withholding water for 20 d, and the survival rates were calculated 3 d after resuming watering. (B) Measurement of leaf water loss rates. Rosette leaves detached from the plants (4-week-old) were weighed at the designated time points after their excision. Water loss was determined as the percentage of the initial fresh weight. The data represent the means ± SD of 180 leaves.

Considering the mannitol-related phenotype of the *TsApx6*-overexpressing plants (Fig 3), it was plausible that *TsApx6* was involved in the response to drought stress. Thus, the drought tolerance of the *atapx6* mutant, *atapx6/35S*:*TsApx6-GFP*, *35S*:*AtApx6-GFP*, and *35S*:*TsApx6-GFP* genotypes was compared with that of the WT by subjecting plants, which were grown for four weeks under well-watered condition, to drought stress by withholding watering for 20 d. During this period, most WT plants and almost all the mutant lines were seriously withered (Fig 6A). The survival rates were determined 3 d after re-watering. Significantly lower survival rate was observed in the *atapx6* mutants (7.5%) compared to the WT plants (40%) (*P* < 0.01) (Fig 6A). However, a majority of the *atapx6/35S*:*TsApx6-GFP*, *35S*:*AtApx6-GFP*, and *35S*:*TsApx6-GFP* plants recovered from withering after rehydration (*P* < 0.05), and the survival rates of *atapx6/35S*:*TsApx6-GFP*, *35S*:*AtApx6-GFP*, and *35S*:*TsApx6-GFP* plants ranged from 41.7% (50 of 120 for line 1) to 56.5% (65 of 115 for line 6), 76.9% (90 of 117 for line 12) to 82.5% (99 of 120 for line 8) and 75.4% (89 of 118 for line 2) to 83.3% (100 of 120 for line 4), respectively (Fig 6A). These results suggest that the overexpression of *TsApx6* enhances drought tolerance of the plants, while loss-of-function of *atapx6* compromised drought tolerance. Thus, *TsApx6* promotes drought tolerance of plants.

Transpiration water loss rates in the detached rosette leaves collected from the plants 4-week-old were further determined at room temperature with a humidity of 50–60%. The results indicated that the leaves from the *atapx6* mutant and the WT plants exhibited a higher rate of water loss than the overexpressing genotypes (*P* < 0.01). The fresh weights of the *atapx6* mutant and the WT leaves after 5-h of incubation were 24% and 42% of their starting values (*P* < 0.01) (Fig 6B). In contrast, the water contents of the *Apx6* overexpressors were approximately 53% (*atapx6/35S*:*TsApx6-GFP*) to 64% (*35S*:*TsApx6-GFP* and *35S*:*AtApx6-GFP*), and the difference between *atapx6/35S*:*TsApx6-GFP* and *35S*:*TsApx6/AtApx6-GFP* lines was significant (*P* < 0.01) (Fig 6B). These results further indicate that the *Apx6* provides the transgenic plants higher level of drought tolerance compared to the wild-type.

### Transcriptional alterations of stress/ABA-responsive genes by *TsApx6*

**Fig 7:**
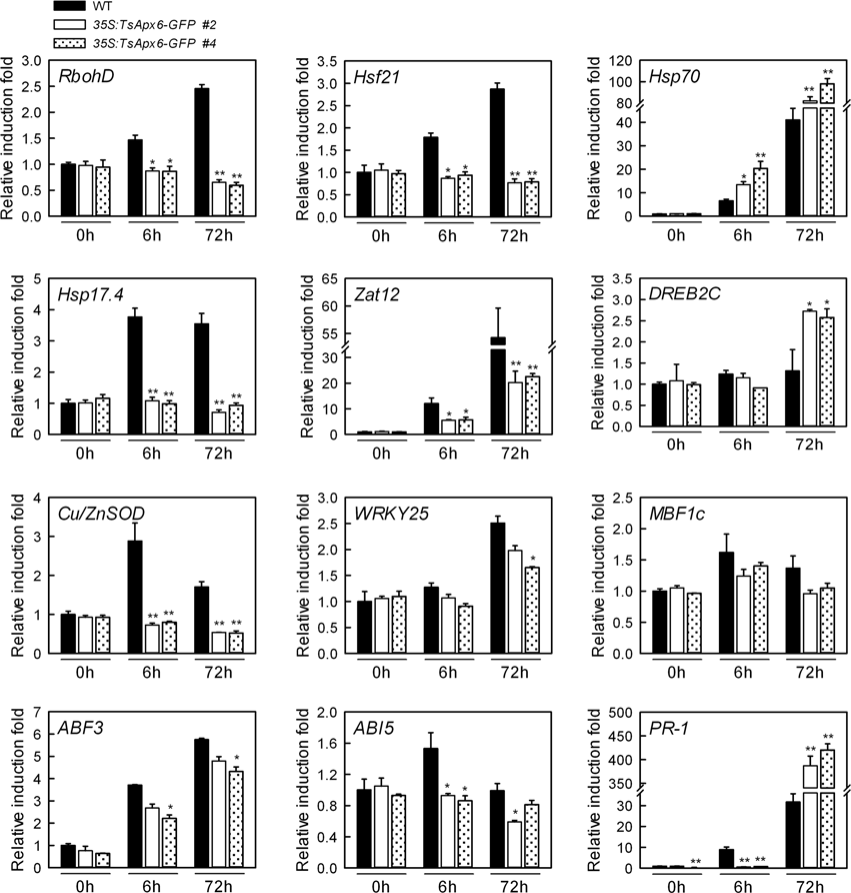
Expression of stress/ABA-responsive genes in the wild-type and the *35S*:*TsApx6-GFP* plants subjected to salt stress. Total RNA was purified from plants (4-week-old) treated with 300 mM NaCl for 6 h or 72 h and subjected to qPCR analysis. The expression of a gene in the WT grown under the control conditions was regarded as 1. The transcript abundance was normalized to the expression levels of *Actin2* gene. Data represent means ± SD (n = 3) from three technical replicates. Asterisks indicate significant difference from the corresponding WT (*: *P* < 0.05; **: *P* < 0.01).

To further study the mechanisms of the changed resistance of *35S*:*TsApx6-GFP* overexpressing plants to stress, we assayed the expression patterns of several genes that were involved in the stress- and ABA- responsive processes under normal and salt stress conditions. After pretreatment with 300 mM NaCl for 6 h or 72 h, the expression levels of *RbohD* (GenBank Accession No. AF055357), *Cu/ZnSOD* (Accession No. EF408820), *Hsp70* (Accession No. NM_112093), *Hsp17*.*4* (Accession No. NM_114492), *Hsf21* (Accession No. NM_118004), *Zat12* (Accession No. NM_125374), *DREB2C* (Accession No. NM_129594), *WRKY25* (Accession No. AF418309), *MBF1c* (Accession No. NM_113358), *ABF3* (Accession No. AK175851), *ABI5* (Accession No. NM_129185) and *PR-1* (Accession No. NM_127025) were analyzed by qPCR. The transcript abundance of *Hsp70*, *DREB2C* and *PR-1* was significantly higher in the *TsApx6*-overexpressing *Arabidopsis* plants than the WT under a 72-h salt stress condition (*P* < 0.05) (Fig 7). However, the fold changes of *RbohD*, *Hsf21*, *Hsp17*.*4*, *Zat12*, *Cu/ZnSOD*, *ABF3* and *ABI5* were significantly lower in the *35S*:*TsApx6-GFP* lines than those in the WT (*P* < 0.05). The expression level of *WRKY25* in the *TsApx6*-overexpressing lines was higher than that in the WT under longer duration of salt stress (*P* < 0.05), whereas no significant change in the *MBF1c* transcript was observed in both WT and *35S*:*TsApx6-GFP* plants under salt stress conditions (Fig 7).

### Isolation and bioinformatics analysis of the *TsApx6* promoter

The upstream flanking region of *TsApx6* 1,654 bp in length was isolated from *T*. *salsuginea* by the chromosome walking method and specific primers (Table A in S1 Text). Sequence of the fragment shared about 89% similarity to the same size upstream region of *AtApx6* gene (S3 Fig). Several putative *cis*-acting regulatory elements were identified within the amplified fragment using the online software PLANTCARE (Table C in S1 Text). They contained different elements that were associated with hormone and stress-related responses: GARE-motif (gibberellin-responsive), CGTCA-motif (MeJA-responsiveness), LTR (low-temperature), ACE (light responsiveness), and ARE (anaerobic induction) (Table 1). Among them, MBS element was specific to the *TsApx6* promoter when compared to the promoter sequence of *AtApx6* (S4 Fig).

**Table 1:** Putative *cis*-elements present in the promoter sequence of *TsApx6*.

Putative *cis*-element	Motif sequence	Function
CGTCA-motif	CGTCA	Involved in MeJA-responsiveness
GARE-motif	TCTGTTG	Gibberellin-responsive element
LTR	CCGAAA	Involved in low-temperature
MBS	CGGTCA	MYB binding site involved in drought inducibility
TC-rich repeats	ATTTTCTTCA	Involved in defense and stress responsiveness
ACE	GCGACGTACC	Involved in light responsiveness
TGA-element	AACGAC	Auxin-responsive element
ARE	TGGTTT	Essential for the anaerobic induction

### Tissue-specific and stress-responsive expression patterns of *TsApx6*

**Fig 8:**
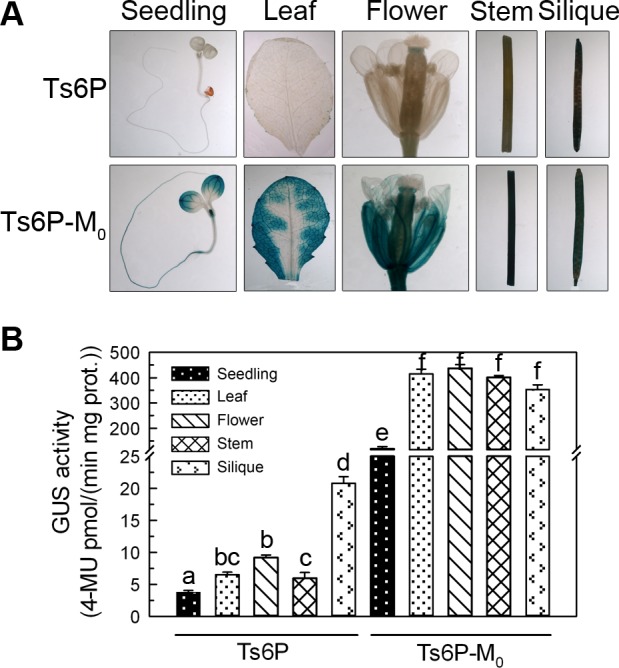
Histochemical staining and GUS assay of transgenic *Arabidopsis* lines Ts6P and Ts6P-M_0_ in different tissues. (A) Histochemical staining of seedlings, leaves, flowers, stems and siliques. (B) GUS activities of the tissues from Ts6P and Ts6P-M_0_ transgenic plants. A significant difference (*P* < 0.05) among the tissues is shown by different letters (a-f).

To determine the full-length *TsApx6* promoter (designated Ts6P) expression pattern and to identify the effect of MBS *cis*-element in this promoter, histochemical staining was carried out at different developmental stages of Ts6P and Ts6P-M_0_ (mutation of the MBS element) plants. The activity of GUS was only weakly observed in the seedlings, leaves, flowers and stems of Ts6P plants. However, when the sequence of MBS element was mutated, the intensity was dramatically increased, and strong GUS signal was observed throughout the entire plant (Fig 8A). Interestingly, GUS activity was greater in the siliques than in the other tissues of the Ts6P plants (*P* < 0.01). In contrast, no significantly difference of GUS expression was observed in the whole mature Ts6P-M_0_ plants (Fig 8B).

**Fig 9:**
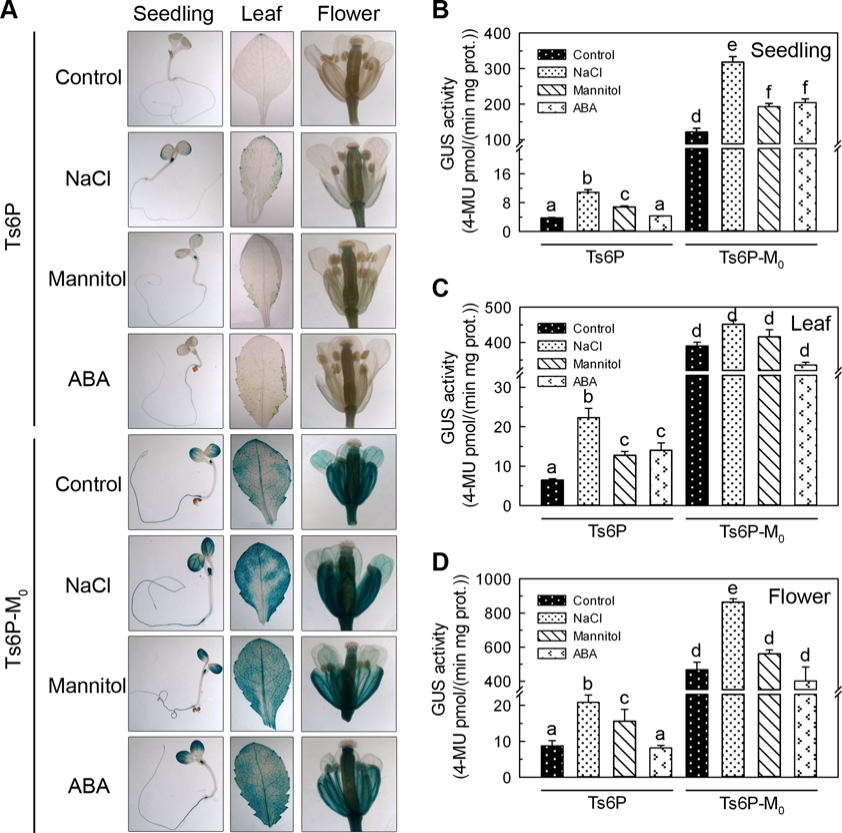
Histochemical staining and GUS assay of transgenic *Arabidopsis* lines Ts6P and Ts6P-M_0_ under different stress conditions. (A) Histochemical staining of seedlings, leaves and flowers treated with 200 mM NaCl, 300 mM mannitol and 0.1 mM ABA for 10 h, respectively. (B) GUS enzymatic activity quantification of the transgenic tissues treated with different stresses. A significant difference (*P* < 0.05) among the tissues is shown by different letters (a-f).

After treatment with 200 mM NaCl, 300 mM mannitol or 0.1 mM ABA for 10 hours separately, strong GUS activity was detected in the seedlings, leaves and flowers of the Ts6P-M_0_ plants, but was weekly detected in the same tissues of the Ts6P plants under the same control and stress conditions (Fig 9A). In the presence of salt stress treatment, the GUS activity in the seedlings and flowers was induced 2.89, and 2.38 folds in the Ts6P plants (*P* < 0.01), while it was increased about 2.63, and 1.85 folds in the same tissues of the Ts6P-M_0_ plants (*P* < 0.05) (Fig 9B and 9D). There was no significant effect of NaCl on the GUS activity in the leaves of Ts6P-M_0_ plants (Fig 9C). In the presence of mannitol, stronger induction of GUS activity was detected in the Ts6P plants, especially in the leaves and flowers, compared to the Ts6P-M_0_ plants (*P* < 0.05). Abscisic acid had no obvious and consistent effect on the GUS signal in the seedlings, leaves and flowers of the two lines (Fig 9B–9D). All of the results indicated that *TsApx6* may take part in plant abiotic stress responses, in particularly, salt and dehydration responses.

## Discussion

The present study focused on an ascorbate peroxidase, which is located in cytoplasm and has important function in the physiological and metabolism systems in *T*. *salsuginea*. A strong induction of *TsApx6* expression by salt stress was observed, which suggests that it might participate in the stresses tolerance. A variety of phenotypes with resistance to stresses were obtained in the *TsApx6* transgenic plants. Root length, germination rate, and cotyledon greening of the *TsApx6* overexpressing *Arabidopsis* were remarkably increased compared to the WT on the NaCl or mannitol containing MS medium. When water was withdrawn from plants (four-week-old) for 20 d, the survival rates for the transgenic plants were greater than those of the WT. The water loss of the detached rosette leaves collected from *TsApx6-*overexpressing lines was slower than that of the WT. Generally, malondialdehyde concentration is used as an indicator for oxidative damage that is induced by salt stress [29]. One of the mechanisms underlying tolerance to salinity is alleviating oxidative damage and mainlining the integrity of the cellular membranes under salt stress [30]. Proline is the most common osmolyte that accumulates in plants in the response to different stresses. Proline performs a wide range of protective functions, for example, osmotic adjustment, cellular structure stabilization and photosynthetic apparatus damage reduction [31]. In this study, the MDA and proline concentrations in the *TsApx6*-overexpressing lines were significantly lower than those in the WT plants under salt stress treatment. However, excessive accumulation of ROS results in extensive damage to plants [32], and SOD, APX, CAT and GPX are major antioxidant enzymes that are used to scavenge ROS for remedying the harm to plants [26]. The concentration of H_2_O_2_ was significantly lower and the APX, GPX, CAT and SOD activities were significantly higher in the *35S*:*TsApx6-GFP* transgenic plants than in the WT plants in the presence of salt stress, indicating that *TsApx6* might contribute to the coordinated upregulation of ROS eliminating enzymes and the lower level of H_2_O_2_. In addition, the NaCl- and mannitol-sensitive phenotype of *atapx6* was sufficiently complemented by the expression of *TsApx6*. These results strongly suggest that TsAPX6 is a key participator in the tolerance to salt stress and water deficit.

As expected, the expression levels of *RbohD*, *Hsf21*, *Hsp70*, *Hsp17*.*4*, *Zat12*, *DREB2C*, *Cu/ZnSOD*, *WRKY25*, *MBF1c*, *ABF3*, *ABI5* and *PR-1* were up-regulated by salt stress. *RbohD* encodes a NADPH oxidase, which promotes ROS production especially after stress treatment [33], and *Cu/ZnSOD* is an important plant ROS scavenger under stress conditions [34]. In this study, the expression of *RbohD* and *Cu/ZnSOD* was significantly lower in the *TsApx6* transgenic plants than in the WT, which might contribute to the lower H_2_O_2_ content and stronger salt resistance of the *TsApx6*-overexpressing plants. HSF21, ZAT12, DREB2C, WRKY25 and MBF1c are the key transcription factors, and they can promote many stress-responsive genes expression under different stress treatments, such as drought, salt, heat or other conditions [14,35–38]. Only *DREB2C* showed a significantly higher expression induction in *TsApx6*-overexpressing plants compared to the WT under salt stress treatment, indicating an indispensable effect on enhanced stress resistance of *TsApx6*-overexpressing plants. HSPs possessed molecular chaperone activities and were the key factors contributing to plants under both normal and adverse growth conditions [39]. The induction of *Hsp70* in the *35S*:*TsApx6-GFP* transgenic plant was significantly higher but the *Hsp17*.*4* was lower than in the WT plants after NaCl treatment, suggesting that *Hsp70* in the *TsApx6*-overexpressing plants was a significant participant in the salt stress condition. Genes *ABI5* and *ABF3* are vital components in the ABA signaling pathway, but the transcript levels of both genes were lower in the *35S*:*TsApx6-GFP* transgenic lines than in the WT, indicating that *TsApx6* modulated salt stress responses independent of the ABA signaling pathway. Surprisingly, the expression of *PR-1*, which encodes a pathogenesis-related protein and can be strongly induced in response to pathogen infections, was also much higher in *TsApx6*-overexpressing lines than in the WT. This suggests that *TsApx6* is involved in a complicated network of regulatory stress responses [40].

A comparative study of the abiotic response between *TsApx6* and *AtApx6* was made in this study. The drought tolerance and dehydration rates of the *35S*:*TsApx6-GFP* and *35S*:*AtApx6-GFP* lines were similar. This suggests that the expression patterns of TsAPX6 and AtAPX6 were different under salt stress despite of the same biochemical function. The mRNA levels of *TsApx6* significantly increased in response to the salt stress, whereas the *AtApx6* transcripts showed no noticeable change. Moreover, analysis by semi-quantitative reverse transcription PCR (RT-PCR) indicated that the *TsApx6* expression was higher than the *AtApx6*, which was almost undetectable under normal growth conditions (S5 Fig). Based on these findings, *TsApx6* may play an important role in both the response to salt stress and the development of *T*. *salsuginea*, and the regulation of the APX gene expression in *T*. *salsuginea* is different from that in *Arabidopsis* under salt stress.

To understand the reason for the different expression patterns of *TsApx6* and *AtApx6* in plant development and abiotic stress response, we analyzed the promoter sequences of these genes. As expected, the two promoter sequences shared 89% sequence similarity (S3 Fig). Bioinformatics analysis showed a difference of the *cis*-acting elements in the two promoters (S4 Fig). The expression levels of *Gus* gene driven by both the *TsApx6* and the *AtApx6* promoters at all developmental stages were very low, but the former was slightly higher than the latter (S6 Fig), which was consistent with our previous work (S5 Fig). In addition, *Gus* expression in Ts6P lines showed greater inducibility than did in At6P lines under abiotic stress conditions (S7 Fig). All these demonstrated that the difference in expression patterns of the *TsApx6* and *AtApx6* might be attributed to the different types and distribution of the *cis*-acting elements in the two promoters.

MBS motif was specific to the *TsApx6* promoter when compared to the promoter sequence of *AtApx6* and predicted to be a MYB binding site that was involved in drought inducibility. To study the function of the MBS in detail, we constructed the Ts6P-M_0_ promoter-reporter vector in which the MBS motif was mutated. To our surprise, the GUS activities in the Ts6P-M_0_ plants were significantly higher than that of the Ts6P at different development stages and under the stress conditions. This result indicated that the MBS motif might be a negative regulatory element and indispensable to the expression regulation of *TsApx6* in development and stress response. Further study on this region to identify the protein that interacts with the MBS motif will be useful to reveal the regulation mechanism of *TsApx6*.

In short, this work provides important understanding of the function of *TsApx6* and regulatory properties of the *TsApx6* promoter. TsAPX6 was an effective H_2_O_2_-scavenging enzyme and the overexpression of *TsApx6* in *Arabidopsis* protected plants from high salinity and water deficit stresses. The expression of *TsApx6* was significantly induced by the abiotic stresses, such as salt, dehydration, and ABA treatments. Moreover, the MBS motif was confirmed as a transcription silencer and it might be important in the regulation of *TsApx6* expression in development and stress response in plants.

## Supporting Information

S1 Fig Acquirement of Ts6P-M_0_ in which the MBS motif was mutated. (A) Electrophoresis of the mutated fragment. (B) Mutated site of the MBS motif.
(TIF)

S2 Fig Sequence obtained and analysis of *TsApx6*. (A) DNA electrophoresis of *TsApx6*. (B) Phylogenetic analysis of the DNA sequences of *TsApx6* and *Arabidopsis Apx* family members. (C) Phylogenetic analysis of the amino acid sequences of *TsApx6* and the members in the *Arabidopsis Apx* family.
(TIF)

S3 Fig Alignment of predicted DNA sequences of *TsApx6* and *AtApx6* promoters. (TIF)

S4 Fig Comparison of the *cis*-acting elements predicted in the *TsApx6* promoter to the corresponding sequence of the *AtApx6* promoter. (A) *cis*-acting elements predicted in *AtApx6* promoter. (B) *cis*-acting elements predicted in *TsApx6* promoter.
(TIF)

S5 Fig Semi-quantitative RT-PCR analyses of *TsApx6* and *AtApx6* expression under the salt stress conditions. Six-week-old *Thellungiella* and four-week-old *Arabidopsis* plants were treated with 300 mM NaCl for 0, 2, 4, 6, 8, 10, 12, 24, 36, or 48h, respectively. Concentrations of RNA from different samples were accurately quantified prior to synthesis of cDNA.
(TIF)

S6 Fig Histochemical staining and GUS assay of transgenic *Arabidopsis* lines At6P and Ts6P in different tissues. (A) Histochemical staining of At6P seedlings, leaves, flowers, stems and siliques. (B) Histochemical staining of Ts6P. (C) Expression of *Gus* from At6P and Ts6P tissues. Relative expression levels were determined with respect to the expression of *Actin2*, whose expression level was defined as 100 relative expression units (REU). (D) GUS activities of the tissues from At6P and Ts6P transgenic plants.
(TIF)

S7 Fig Histochemical staining and GUS assay of transgenic *Arabidopsis* lines At6P and Ts6P under different stress conditions. (A) Histochemical staining of seedlings, leaves and flowers treated with 200 mM NaCl, 300 mM mannitol, and 0.1 mM ABA for 10 h, respectively. (B, D and F) Expression of *Gus* from At6P and Ts6P tissues treated with different stresses. Relative expression levels were determined with respect to the expression of *Actin2* (= 100 REU). (C, E and G) Quantification of GUS enzymatic activity in the transgenic tissues treated with different stresses.
(TIF)

S8 Fig Expression of *Gus* gene from Ts6P and Ts6P-M_0_ plants in different tissues or under different stress conditions. Relative expression levels were determined with respect to the expression of *Actin2* (= 100 REU).
(TIF)

S1 Text Primers used for gene expression assays and sequence and predicted *cis*-acting elements of the *TsApx6* promoter. (Table A) Primers used for gene expression assays. (Table B) Sequence of *TsApx6* promoter. (Table C) Predicted *cis*-acting elements of the *TsApx6* promoter.
(DOCX)
