# Supplementary figures and images for: The Functional and Regulatory Mechanisms of the Thellungiella salsuginea Ascorbate Peroxidase 6 (TsAPX6) in Response to Salinity and Water Deficit Stresses
Source: PLoS One. 2016 Apr 20;11(4):e0154042. doi: 10.1371/journal.pone.0154042 (PMC4838305; doi:10.1371/journal.pone.0154042)

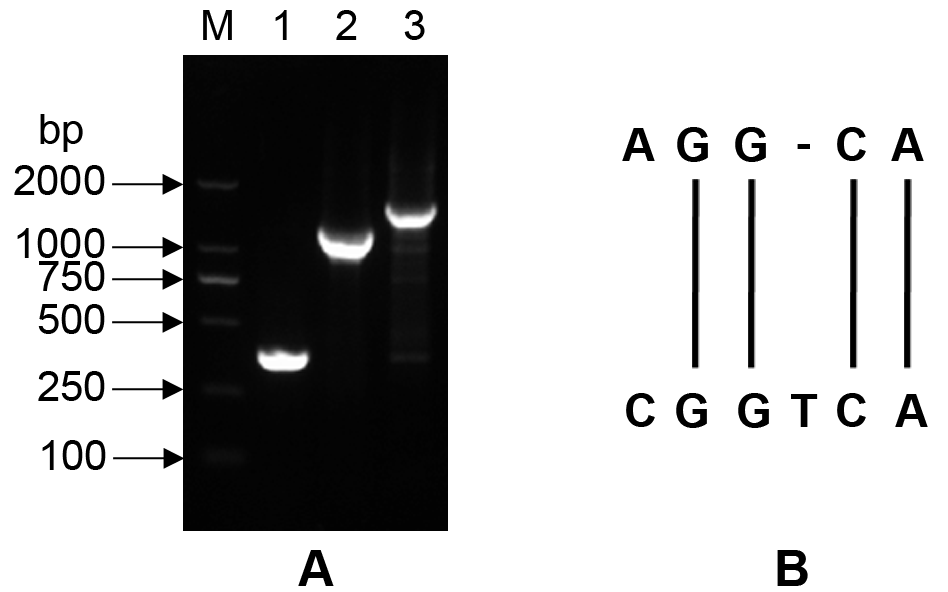

Supplement: S1 Fig — (A) Electrophoresis of the mutated fragment. (B) Mutated site of the MBS motif. (TIF) [file pone.0154042.s001.tif]

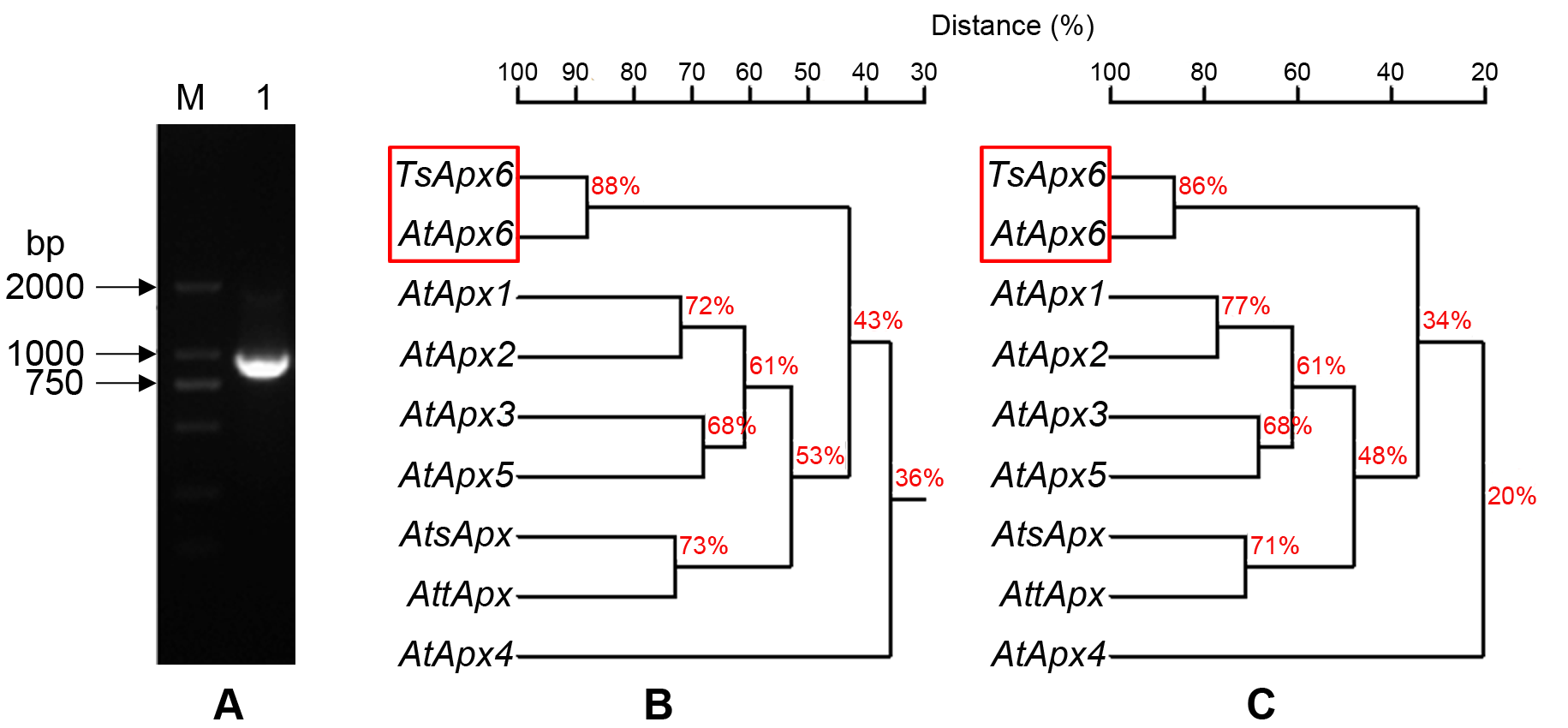

Supplement: S2 Fig — (A) DNA electrophoresis of TsApx6. (B) Phylogenetic analysis of the DNA sequences of TsApx6 and Arabidopsis Apx family members. (C) Phylogenetic analysis of the amino acid sequences of TsApx6 and the members in the Arabidopsis Apx family. (TIF) [file pone.0154042.s002.tif]

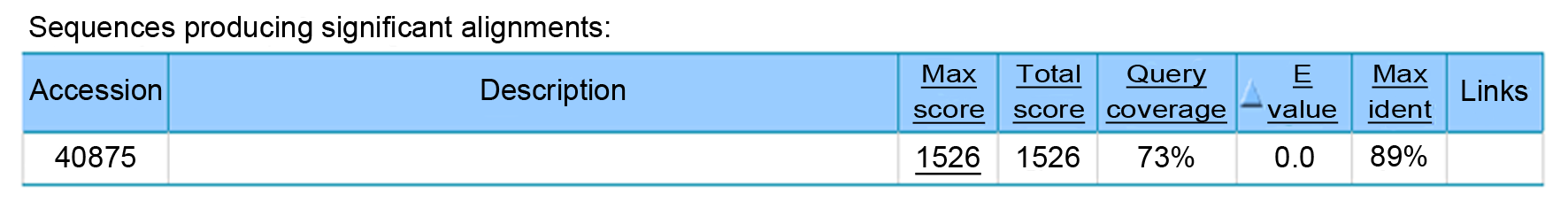

Supplement: S3 Fig — (TIF) [file pone.0154042.s003.tif]

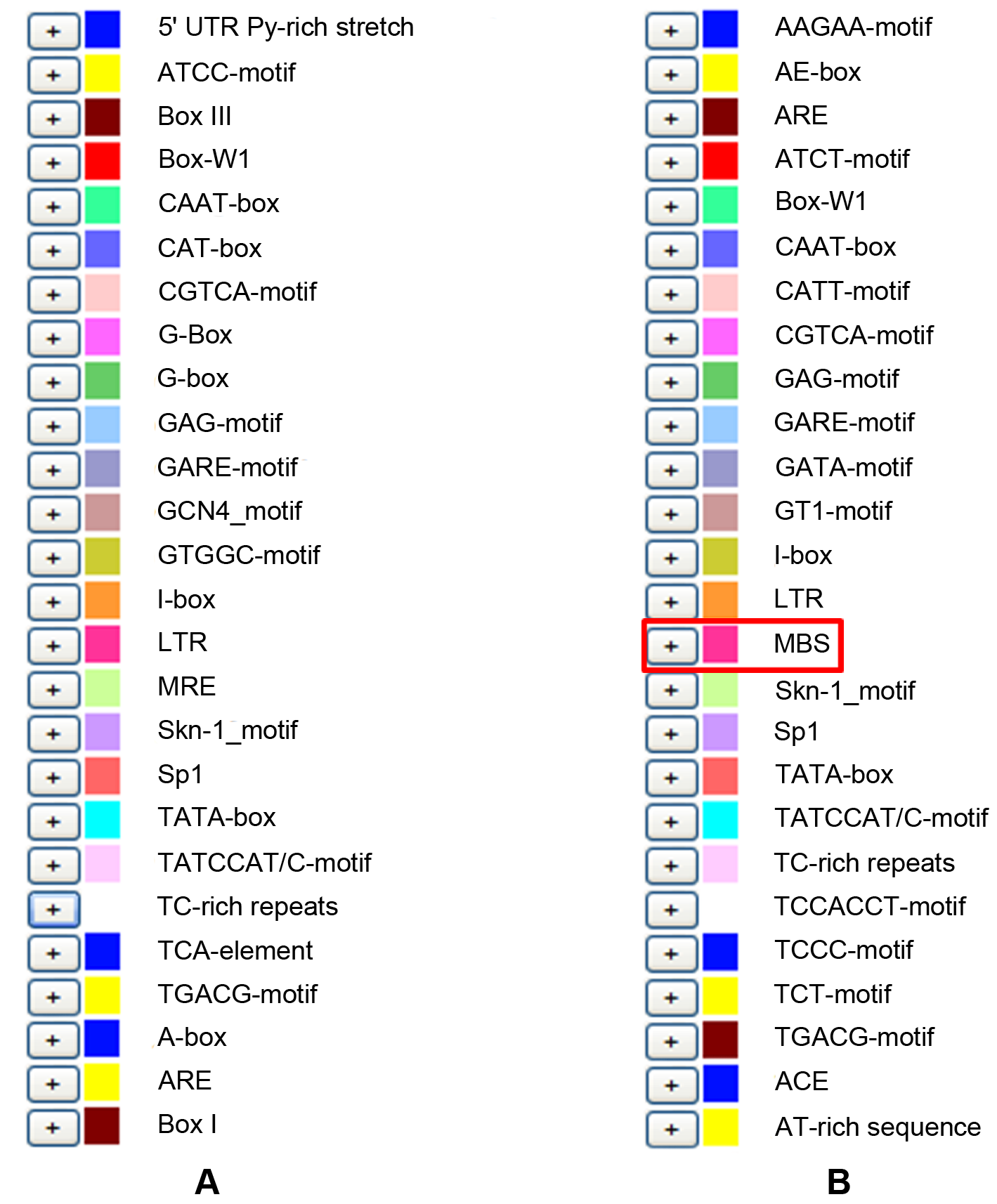

Supplement: S4 Fig — (A) cis-acting elements predicted in AtApx6 promoter. (B) cis-acting elements predicted in TsApx6 promoter. (TIF) [file pone.0154042.s004.tif]

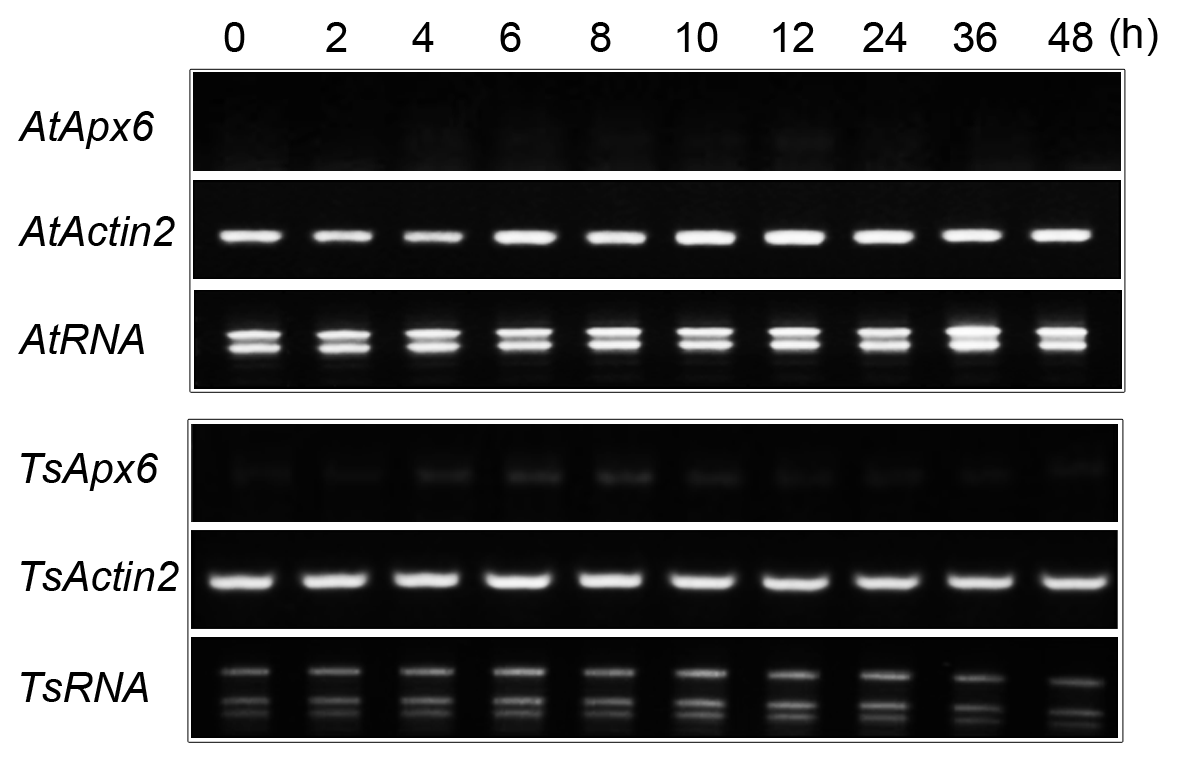

Supplement: S5 Fig — Six-week-old Thellungiella and four-week-old Arabidopsis plants were treated with 300 mM NaCl for 0, 2, 4, 6, 8, 10, 12, 24, 36, or 48h, respectively. Concentrations of RNA from different samples were accurately quantified prior to synthesis of cDNA. (TIF) [file pone.0154042.s005.tif]

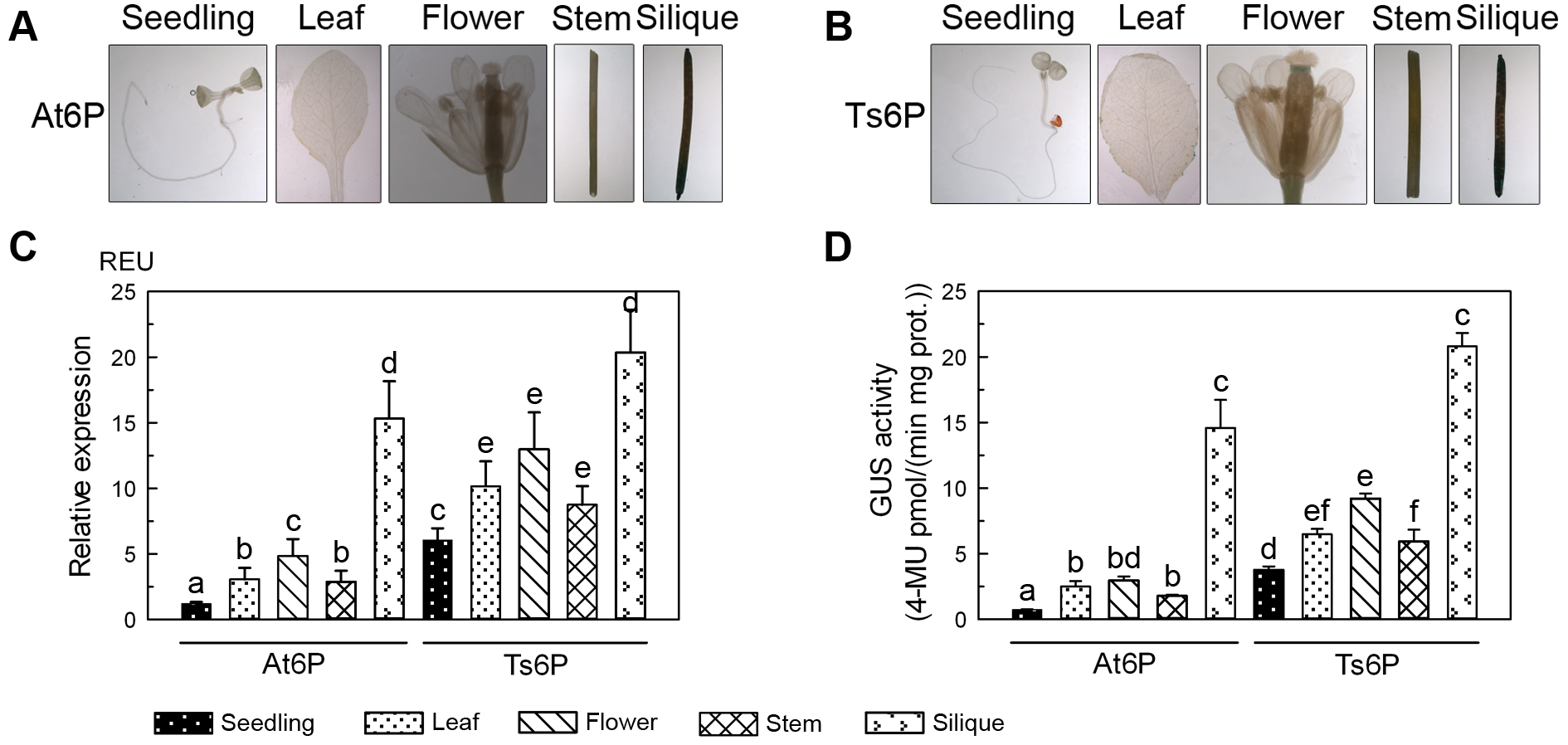

Supplement: S6 Fig — (A) Histochemical staining of At6P seedlings, leaves, flowers, stems and siliques. (B) Histochemical staining of Ts6P. (C) Expression of Gus from At6P and Ts6P tissues. Relative expression levels were determined with respect to the expression of Actin2, whose expression level was defined as 100 relative expression units (REU). (D) GUS activities of the tissues from At6P and Ts6P transgenic plants. (TIF) [file pone.0154042.s006.tif]

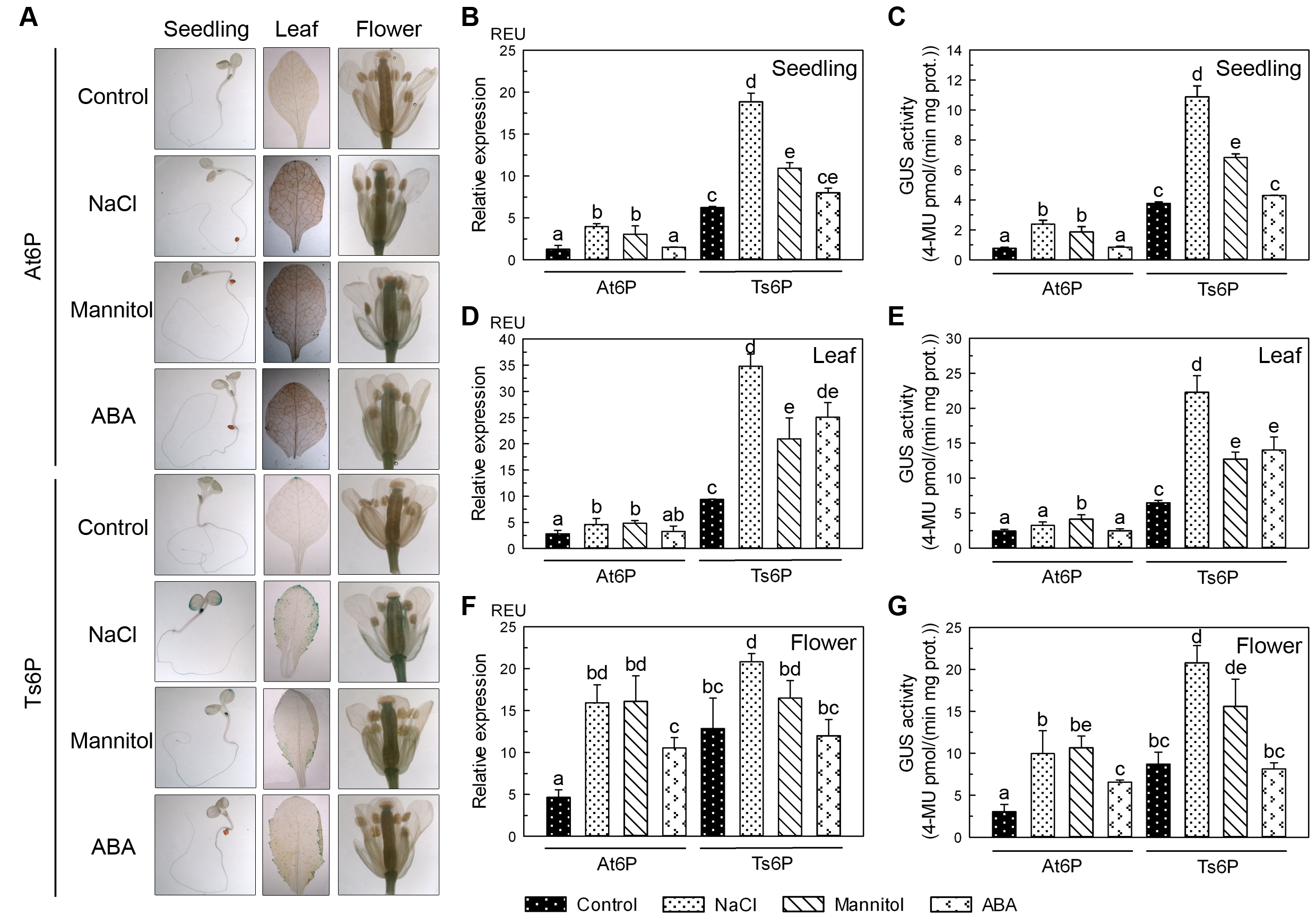

Supplement: S7 Fig — (A) Histochemical staining of seedlings, leaves and flowers treated with 200 mM NaCl, 300 mM mannitol, and 0.1 mM ABA for 10 h, respectively. (B, D and F) Expression of Gus from At6P and Ts6P tissues treated with different stresses. Relative expression levels were determined with respect to the expression of Actin2 (= 100 REU). (C, E and G) Quantification of GUS enzymatic activity in the transgenic tissues treated with different stresses. (TIF) [file pone.0154042.s007.tif]

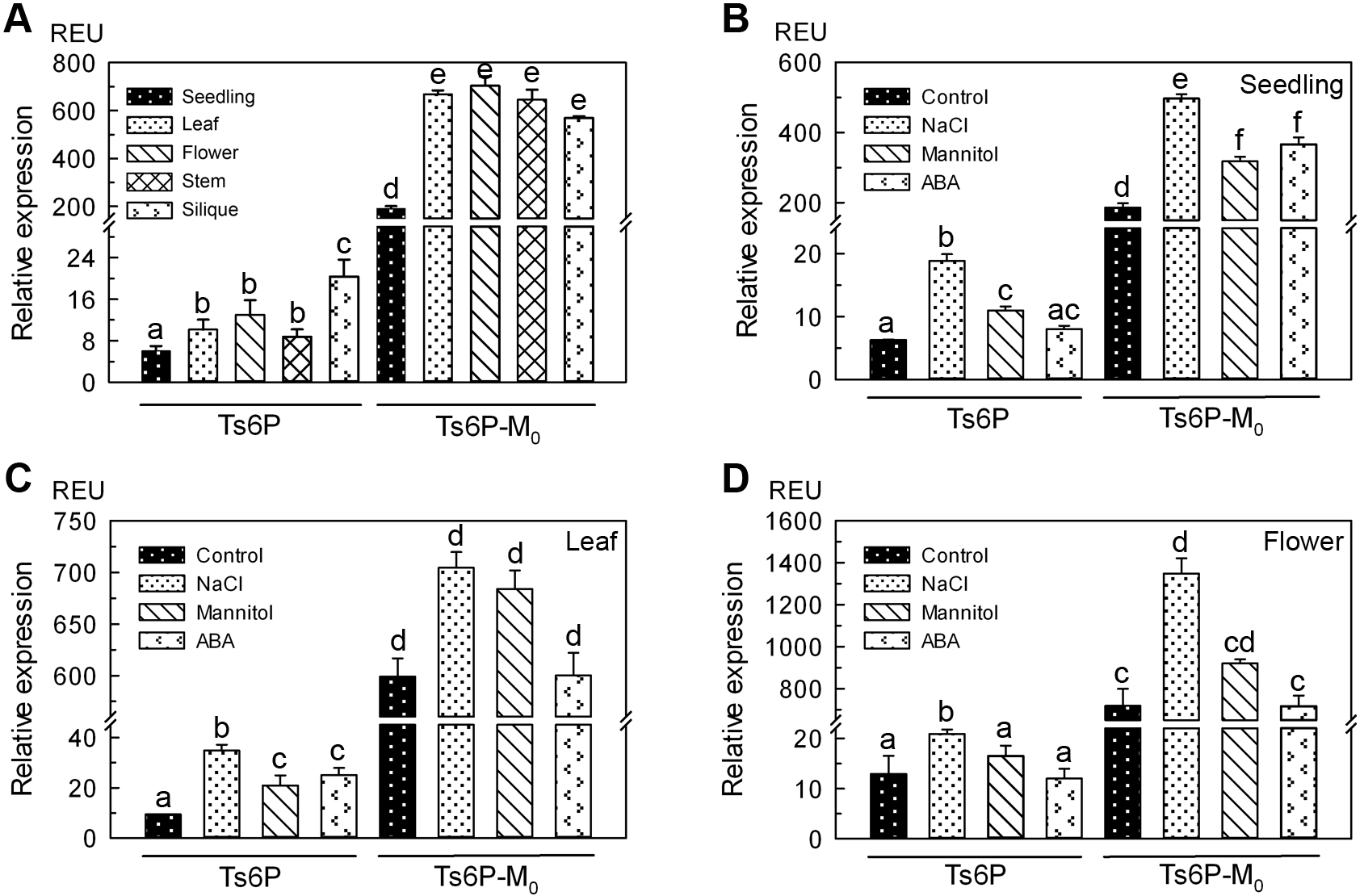

Supplement: S8 Fig — Relative expression levels were determined with respect to the expression of Actin2 (= 100 REU). (TIF) [file pone.0154042.s008.tif]
