# Supplementary material for: The Functional and Regulatory Mechanisms of the Thellungiella salsuginea Ascorbate Peroxidase 6 (TsAPX6) in Response to Salinity and Water Deficit Stresses
Source: PLoS One. 2016 Apr 20;11(4):e0154042. doi: 10.1371/journal.pone.0154042 (PMC4838305; doi:10.1371/journal.pone.0154042)
Supplement: S1 Text — (Table A) Primers used for gene expression assays. (Table B) Sequence of TsApx6 promoter. (Table C) Predicted cis-acting elements of the TsApx6 promoter. (DOCX) [file pone.0154042.s009.docx]

**Table A. Primers used for gene expression assays**

| Primer name | Primer sequence (5'→3') | Purpose |
| --- | --- | --- |
| *Apx6*-LP_c_ | ATGACGACGACGACTGCTTCT | Cloning of *TsApx6* and *AtApx6* |
| *Apx6*-RP_c_ | TCATAACATATTCCATTTGGCCC |  |
| *Apx6*-LP | TGCAAAACGAAATAAGGAAAGTGGTG | qRT-PCR of *TsApx6* and *AtApx6* |
| *Apx6*-RP | CACTCAGGGTTTCTGGAGGTAGCTTG |  |
| *Actin2*-LP | GTCCATTCGGAACAATGAGGTTTGAC | qRT-PCR of *Actin2* |
| *Actin2*-RP | GTGGGCACCAGATAAAGCGACAAT |  |
| *Apx6PstI*-LP | AACTGCAGATGACGACGACGACTGCTTCT^1^ | Cloning *Apx6* into pCAMBIA1302 |
| *Apx6AvrII*-RP | ATCCTAGGTCATAACATATTCCATTTGGCCC^2^ |  |
| T*Apx6*-LP | TGGGTCTGTAGCGGTTTCAATA | Confirming of *Apx6* in transgenic plants |
| T*Apx6*-RP | AGGTCAGGGTGGTCACGAGGGT |  |
| *HsfA4a*-LP | AGAAGCGTCAGAAAACAAT | Expression assay of stress/ABA-responsive genes |
| *HsfA4a*-RP | TCACAAGAATCCGATACAA |  |
| *PR-1*-LP | TTCACAACCAGGCACGA |  |
| *PR-1*-RP | AAGTCACCGCTACCCCA |  |
| *ABF3*-LP | AGGAGTTTTTGGTCAGGG |  |
| *ABF3*-RP | TGTATCAGTTGTTGCGGC |  |
| *ABI5*-LP | ATAAGAGAGGGATAGCGA |  |
| *ABI5*-RP | AGGTTTAGGATTAGTGGG |  |
| *WRKY25*-LP | GAAGAAGAGCGAAAACCC |  |
| *WRKY25*-RP | TTCCTCCATCTAAAGCCA |  |
| *Hsp17.4*-LP | CAAACGCACCTGCCAAG |  |
| *Hsp17.4*-RP | AACCGACAACACCCCAT |  |
| *Hsp70*-LP | TAAGCGTCTAATCGGAAG |  |
| *Hsp70*-RP | CGTTGAAATAAGCAGGAA |  |
| *Zat12*-LP | GTCACAAGAAGCCTAACAAC |  |
| *Zat12*-RP | CCCTAGACTCAGATCCAAAC |  |
| *RbohD*-LP | CAATAGTGTTGCTGGCG |  |
| *RbohD*-RP | GAGGCGTTCTTGATGCG |  |
| *MBF1c*-LP | CCCAGGAGCAGTAACA |  |
| *MBF1c*-RP | GAACCGCCGTAGATTT |  |
| *DREB2C*-LP | AATCCGAAAGCCTCCT |  |
| *DREB2C*-RP | GCAACCCATTTACCCC |  |
| *Cu/ZnSOD*-LP | AGTGAGGGTGTTACGGGGAC |  |
| *Cu/ZnSOD*-RP | ATCGGGGTTGAAATGTGGAC |  |
| Ts6P-1SP1 | GCCTTTCCCTTTGTCACCACTT | Chromosome walking for the first time |
| Ts6P-1SP2 | GCATCGTGGACAACAGAACAAC |  |
| Ts6P-1SP3 | TCGGATAACATGATTCCCAGTC |  |
| Ts6P-2SP1 | TACCACCACCTTCCTCGCTAAT | Chromosome walking for the second time |
| Ts6P-2SP2 | CAAAGTGGAAGAAGTCGGAGGC |  |
| Ts6P-2SP3 | TTTCATCGCCCTTTGCTTCTGC |  |
| Ts6P-LP | GCGTCGACTCGTGTCTTACATCAAGGTGGCA^3^ | Cloning of full-length *TsApx6* promoter |
| Ts6P-RP | AGACTAGTAGACTAGTCTTTAACGGTGGGACGAG^4^ |  |
| At6P-LP | GCGTCGACTTGCTCGTGCCTTGGTCTTTTC^3^ | Cloning of full-length *AtApx6* promoter |
| At6P-RP | AGACTAGTTCATCTTTAACGGTGGGACGAG^4^ |  |
| Ts6P-LPa | TCGTGTCTTACATCAAGGTGG | Cloning of Ts6P-M_0_ promoter |
| Ts6P-BPb | CTTGCCTATTCCTTCAAGTTTC |  |
| Ts6P-BPc | AGGCAAGAAAGAATTCAGAGCCG |  |
| Ts6P-RPd | CTTTAACGGTGGGACGAG |  |
| *gus*-LP | TGGACTACGGGAAAGGAC | Expression assay of *gus* in transgenic plants |
| *gus*-RP | GCCGAAATCTGGAATGTT |  |

^1,2,3,4^ Underlined sequences, recognition sites for ^1^*Pst* I, ^2^*Avr* II, ^3^*Sal* I, and ^4^*Spe* I

**Table B. Sequence of *TsApx6* promoter (Ts6P, 1654 bp)**

| -1654 | TCGTGTCTTA | CATCAAGGTG | GCAACTAATG | GTGCGGGAGG | TGGAGATAAG |
| --- | --- | --- | --- | --- | --- |
| -1604 | GGAGAAGATG | ATGGGAAACA | TTTTCCGTAT | ATAGTGATTA | TTGTAGTGGT |
| -1554 | GACGGTTTTT | ATCATCGGTG | TTTTGATCTT | TGTGGCGTTT | CGGATTCATA |
| -1504 | AGAAGAAGAA | AAAGCTTTTG | GATGATCATC | AAGATCAGAG | TTCAGAGGAA |
| -1454 | GATAACTTCT | TGGAGAATTT | ATCCGGTATG | CCTATCCGGT | TCGCTTACAA |
| -1404 | AGATCTTCAG | TCAGCGACGA | ATAACTTCTC | GGTTAAGTTA | GGGCAAGGAG |
| -1354 | GGTTTGGATC | AGTCTATGAA | GGGACTCTAC | CTGATGGTTC | TCGTTTAGCC |
| -1304 | GTGAAGAAAC | TTGAAGGAAT | **CGGTCA**AGGC | AAGAAAGAAT | TCAGAGCCGA |
| -1254 | GGTTAGTATA | ATCGGAAGCA | TTCATCATCT | GCATTTGGTG | CGGCTCAGGG |
| -1204 | GTTTCTGTGC | GGAAGGGGCT | CATAGGCTTC | TCGCTTATGA | GTTCTTGTCG |
| -1154 | AAAGGTTCGT | TAGAGAGATG | GATATTTAGG | AGAAGAGACG | AAGATATTCT |
| -1104 | GTTGGATTGG | GACACAAGAT | TCAACATCGC | ACTCGGAACA | GCTAAAGGTT |
| -1054 | TAGCGTATCT | ACATGAAGAC | TGCGATGCAA | GAATCATCCA | TTGCGATATC |
| -1004 | AAACCAGAGA | ACATCCTCTT | AGACGATAAC | TTCAATGCCA | AGGTATCTGA |
| -954 | TTTCGGACTC | GCTAAGCTTA | TGACCCGCGA | ACAAAGCCAT | GTATTCACAA |
| -904 | CGATGCGTGG | GACGAGAGGC | TATTTGGCTC | CGGAATGGAT | CACAAACTAC |
| -854 | GCGATCTCGG | AGAAGAGCGA | TGTTTATAGC | TACGGGATGG | TGTTGCTTGA |
| -804 | GCTGATAGGA | GGAAGAAAGA | ACTATGATCC | ATCAGAGACA | TCTGAGAAAT |
| -754 | GCCATTTTCC | TTCTTATGCT | TTCAAGATGA | TGGAAGAAGG | AAAGCTTATG |
| -704 | GAGATTCGAT | TCGAGCTCGG | AACCCGATGA | AGAATCCTCT | GATGTGACTG |
| -654 | AGTGCAGAGA | GTTCAAAGGG | CGATGAAAAC | AGCGCTTTGG | TGTATACAAG |
| -604 | AAGATATGCA | CGCGAGACCT | TCGATGAGCA | AAGTTGTTCA | AATGCTTGAA |
| -554 | GGAGTTTTTC | CGGTGGTTCA | GCCTCCGACT | TCTTCCACTT | TGGGCTCGAG |
| -504 | GCTTTACTCG | AGTTTCTTCA | AGTCGATTAG | CGAGGAAGGT | GGTGGTACGT |
| -454 | CGTCTGGACC | ATCGGATTGT | AACAGTGAGA | ATTATCTCTC | CGCCGTGAGA |
| -404 | CTCTCCGGTC | CGAGATAGCT | CGCTTGTTTT | TAGCCATATA | AAGTATTTTA |
| -354 | TAGTGTTATA | AATAGGTTTT | TTGGCTGTTG | TATATTGTGC | TGTGTGGTAA |
| -304 | AATTTTAAAG | ATTAGTGTCT | CCTCCACTCA | GTTTTGTATT | TTTTAGTTAC |
| -254 | TATAGAATAT | TTGAACTACC | TGTCGGGTTT | CTCTTTTTTC | GTATATCGCC |
| -204 | TTGTCGTTTT | CTATTCTTTT | GTATCGTCAC | GAATTCCGAA | ATGCGTGCCG |
| -154 | TGTCCAAACT | TCTCTCTCTC | TAAATTGTCG | ATTTCTGTGT | TTCTATTGGC |
| -104 | TAGAACATTT | TGCTTACTGT | ACTCTCACGA | GTAGACAATA | CAAACTCCCG |
| -54 | CAGTACATAA | AAAAAATCAG | CTGGCGCTCG | ATGATTCT**C**G | TCCCACCGTT |
| -4 | AAAG**ATG**ACG | ACGACGACCG |  |  |  |

The text highlighted with bold and underline represented, in turn, the sequence of MBS motif, the predicted transcription start site and the translation start site.

**Table C. Predicted *cis*-acting elements of the *TsApx6* promoter**

| Regulatory sequence name | Motif sequence | Position (Strand) | Organism | Function |
| --- | --- | --- | --- | --- |
| CGTCA-motif | CGTCA | -180(+)  -1555(-) | *Hordeum vulgare* | Involved in MeJA-responsiveness |
| TGACG-motif | TGACG | -1553(+), -180(-) | *H. vulgare* | Involved in MeJA-responsiveness |
| GARE-motif | TCTGTTG | -1107(+) | *Brassica oleracea* | Gibberellin-responsive element |
| CAAT-box | CAAT | Number:3(+), Number:11(-) | *Arabidopsis thaliana etc* | Element in promoter and enhancer regions |
| LTR | CCGAAA | -169(+), -1517(-), -954(-) | *H. vulgare* | Involved in low-temperature responsiveness |
| MBS | CGGTCA | -1284(+) | *Zea mays* | MYB binding site involved in drought inducibility |
| TC-rich repeats | ATTTTCTTCA | -494(+), -1303(-) | *Nicotiana tabacum* | Involved in defense and stress responsiveness |
| ACE | GCGACGTACC | -461(-) | *Petroselinum crispum* | Involved in light responsiveness |
| AE-box | AGAAACTT | -1300(+) | *A. thaliana* | Part of a module for light response |
| G-box | GACACGTAGT | -240(-) | *Larix laricina* | Involved in light responsiveness |
| I-box | cGATAAGGCG | -208(-) | *Z. mays* | Part of a light responsive element |
| CATT-motif | GCATTC | -1237(+) | *Z. mays* | Part of a module for light response |
|  | | | | |
| GAG-motif | AGAGATG(GT) | -1141(+), -649(+) | *Spinacia oleracea*,  *A. thaliana* | Part of a light responsive element |
| GATA-motif | GATAGGA | -801(+) | *A. thaliana* | Part of a light responsive element |
| TCT-motif | TCTTAC | -1649(+) | *A. thaliana* | Part of a light responsive element |
| TGA-element | AACGAC | -202(-) | *B. oleracea* | Auxin-responsive element |
| TATA-box | TATA | Number:18(+)  Number:13(-) | *A. thaliana etc* | Core promoter element |
| GT1-motif | GGTTAA | -1374(+) | *A. thaliana* | Light responsive element |
| ARE | TGGTTT | -1004(-) | *Z. mays* | Essential for the anaerobic induction |
| ATCT-motif | AATCTAATCT | -703(-) | *A. thaliana* | Part of a conserved DNA module involved in light responsiveness |
| Box-W1 | TTGACC | -1283(-) | *P. crispum* | Fungal elicitor responsive element |
